# Dual role of the adhesion G-protein coupled receptor ADRGE5/CD97 in glioblastoma invasion and proliferation

**DOI:** 10.1016/j.jbc.2023.105105

**Published:** 2023-07-28

**Authors:** Tatiana I. Slepak, Manuela Guyot, Winston Walters, Daniel G. Eichberg, Michael E. Ivan

**Affiliations:** 1 Department of Neurosurgery, University of Miami Hospital, University of Miami, Coral Gables, USA; 2 Sylvester Comprehensive Cancer Center, University of Miami, Coral Gables, USA

**Keywords:** glioblastoma, adhesion GPCR, tumor invasion, CD97/ADGRE5, cancer stem cells, brain tumor, cell adhesion, tumor microenvironment

## Abstract

CD97, an adhesion G-protein coupled receptor highly expressed in glioblastoma (GBM), consists of two noncovalently bound domains: the N-terminal fragment (NTF) and C-terminal fragment. The C-terminal fragment contains a GPCR domain that couples to Gα_12/13_, while the NTF interacts with extracellular matrix components and other receptors. We investigated the effects of changing CD97 levels and its function on primary patient-derived GBM stem cells (pdGSCs) *in vitro* and *in vivo*. We created two functional mutants: a constitutively active ΔNTF and the noncleavable dominant-negative H436A mutant. The CD97 knockdown in pdGSCs decreased, while overexpression of CD97 increased tumor size. Unlike other constructs, the ΔNTF mutant promoted tumor cell proliferation, but the tumors were comparable in size to those with CD97 overexpression. As expected, the GBM tumors overexpressing CD97 were very invasive, but surprisingly, the knockdown did not inhibit invasiveness and even induced it in noninvasive U87 tumors. Importantly, our results indicate that NTF was present in the tumor core cells but absent in the pdGSCs invading the brain. Furthermore, the expression of noncleavable H436A mutant led to large tumors that invade by sending massive protrusions, but the invasion of individual tumor cells was substantially reduced. These data suggest that NTF association with CD97 GPCR domain inhibits individual cell dissemination but not overall tumor invasion. However, NTF dissociation facilitates pdGSCs brain infiltration and may promote tumor proliferation. Thus, the interplay between two functional domains regulates CD97 activity resulting in either enhanced cell adhesion or stimulation of tumor cell invasion and proliferation.

Glioblastoma (GBM) is the most common type of brain cancer, and it remains incurable. Aggressive invasiveness of this tumor is a hallmark characteristic associated with heterogeneous and highly adaptable cancer cells called glioma stem cells (GSCs) (1, 2, 3, 4).

Multiple studies have been conducted to identify molecular targets in the search for mechanisms that inhibit GBM invasion. Among these targets are the cell surface receptors EGFR, PDGFR, CD44 (5, 6, 7), metalloproteinases MMP14, MMP2, and MMP9 (8), the components of extracellular matrix (ECM) (9, 10, 11), and chemokines released by either tumor cells or microglia (12, 13). These and other studies have illuminated several mechanisms involved in GBM tumor invasion, yet no successful pharmacological inhibitors of GBM invasion have been described thus far. Therefore, additional research is needed to understand the mechanism of GBM invasion and to identify new therapeutic targets.

In this study, we focused on the adhesion G-protein coupled receptor (aGPCR) ADRGE5/CD97 (CD97), which was first identified as a gene highly expressed in lymphocytes and involved in their extravasation during inflammation (14). High levels of CD97 have been detected in gastric (15, 16), prostate (17), pancreatic (18), and other systemic cancers. Its role is associated with increased invasiveness, metastasis, and poor prognosis. In contrast to normal brain tissue and low-grade gliomas, it has been shown that GBM expresses high levels of CD97, which correlates to increased invasiveness and poor survival (19). The same study demonstrated that *in vitro*, the knockdown of CD97 in the GBM U87 cell line reduced invasion but had no effect on GBM proliferation. More recently, transcriptomic analysis of 14 GBM tumors showed that high levels of CD97 are associated with mitogenic pathway activation and changes in the GBM immune microenvironment (20). All functional data on CD97 in these studies was obtained *in vitro* using GBM cell lines. Therefore, the function of CD97 in GBM tumors *in vivo* remains unclear.

CD97 belongs to the epidermal growth factor-seven transmembrane (7-TM) subfamily of aGPCRs (21). Most aGPCRs, including CD97, undergo self-cleavage at the GPCR proteolytic site (GPS) after protein folding. This cleavage produces two fragments that remain noncovalently linked: a very large extracellular N-terminal fragment (NTF) and a smaller 7-TM-GPCR intracellular C-terminal fragment (CTF). The NTF is involved in cellular adhesion *via* its interactions with other cell surface receptors and ECM proteins (17, 22). Several extracellular ligands of CD97 have been identified, including CD55 (DAF) (23), α5β1 integrin (24), CD90 (Thy-1) (25), and chondroitin sulfate (26). Binding of CD97 to these ligands causes a conformational change or dissociation of the NTF, which in turn liberates an internal tethered agonist (TA), a small hydrophobic peptide sequence at the N terminus of the CTF. This leads to TA insertion into the 7-TM-GPCR domain activating Gα_12/13_-dependent pathways (17, 22). In support of this model, the experimental deletion of the NTF (CD97ΔNTF) has been shown to increase the basal activity of the Rho pathway, which affects cancer cell motility and invasion (17, 27).

The unique structure and ability to self-cleave allow CD97 to perform distinct functions that are independently mediated by the NTF and CTF domains. As such, CD97 can be involved in many tumorigenesis processes, including cell adhesion, migration, and invasion (28). Adhesion is important for holding tumor cells together and during attachment to other cells within the tissue. Cancer cell migration and invasion become essential when the growing tumor senses the lack of oxygen and nutrients. MRI images show that GBM cells invade mostly along white matter tracts and blood vessels, with individual cells disseminating throughout the brain (29). Currently, the tumor invasive growth is classified into two distinct types: collective cell migration and single cell migration (30, 31). Collective migration involves interconnected cell groups using adhesion molecules and communication junctions. In GBM, this is exhibited as finger-like protrusions extended from tumor mass, detached cell clusters, or extended cell chords. Collectively invading tumor cells are interconnected by cadherins and integrins, and cells at the leading edge of the protrusions release proteases to degrade the ECM of the surrounding tissue (30). Single cell invasion, on the other hand, is characterized by individual tumor cells independently penetrating surrounding tissue (31). These cells are believed to undergo epithelial-to-mesenchymal transition adopting a mesenchymal phenotype-resembling fibroblasts (32). This fibroblast-like migration is observed in various malignancies, including melanoma, fibrosarcoma, and GBM (33). The detection and elimination of these transformed single GBM cells invading the brain is especially challenging as they are highly resistant to any treatment (34).

In this study, we conducted the first investigation of CD97’s role in GBM tumor growth and invasion *in vivo*. To explore the function of CD97, we utilized primary patient-derived GBM stem cells (pdGSCs) that were genetically modified to manipulate CD97 levels and function. By studying xenografts derived from these modified pdGSCs, we gained novel insights into the impact of CD97 on GBM tumor behavior. This research provides valuable information on the role of CD97 in the context of GBM and expands our understanding of its contribution to tumor invasion and progression.

## Results

### Expression and knockdown of CD97 in pdGSCs

**Figure 1:**
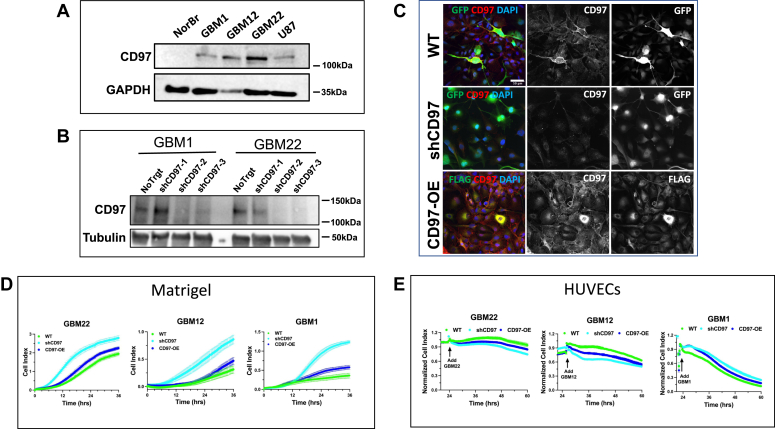
**Effects of CD97 knockdown and overexpression on pdGSCs invasion *in vitro*.***A*, representative immunoblot showing the expression of CD97 in total cell lysates obtained from GBM1, GBM12, GBM22 primary cells, and U87 cell line. The anti-CD97 antibody did not detect CD97 in the noncancerous brain biopsy from a patient with epilepsy (NorBr) used as a negative control. *B*, GBM1 and GBM22 cells were transduced with lentiviruses expressing three different shRNAs against CD97 (shCD97-1-3) or nontarget shRNA (NoTrgt) as a control, as described in Experimental procedures. Cell lysates were prepared 7 days after virus transduction. ShCD97-2 was used in subsequent experiments, and the cells expressing this shRNA were further labeled as shCD97. Immunoblot is a representative of multiple experiments. *C*, immunostaining of GBM22 cells with CD97 antibodies (*red*) shows changes in the endogenous CD97 levels after its knockdown or upon overexpression. GBM22 cells were transduced with control GFP encoding virus (WT), the virus encoding shRNA against CD97 and GFP cDNA (shCD97), or FLAG-tagged CD97 cDNA (CD97-OE). Transduced cells were fixed and stained with antibodies 7 days after puromycin selection. GFP fluorescence (*green*) was used to identify cells expressing control (WT) or CD97 knockdown cells (shCD97). FLAG antibodies (*green*) were used to identify exogenously expressed CD97 in CD97-OE. The confocal images were taken at the same exposure to detect the differences in CD97 expression. Scale bars represent 20 μm. *D*, *in vitro* invasion assay in xCelligence CIM plate was performed as described in Experimental procedures. Cells migrated toward the attractant (10% fetal bovine serum) through the Matrigel supplemented with 20 mg/ml of chondroitin sulfate and 10 μg/ml of hyaluronic acid. Real-time changes in impedance expressed in the arbitrary units of the Cell Index (N = 6, Mean ± SD) were recorded for three primary pdGSCs, GBM22, GBM12, and GBM1. Cells with CD97 knockdown (shCD97, *light blue* traces) crossed the Matrigel faster than CD97-OE cells (*dark blue*) or control (WT, *green*). Shown representative results performed in three independent experiments for each pdGSC. *E*, *in vitro* invasion assay testing the ability of pdGSCs to disrupt HUVECs monolayer. HUVECs were plated at a high density on xCelligence E-plate and cultured overnight until a monolayer was formed as indicated by stability of the Cell Index (flat line). The same GBM primary cells as in (*D*) were added at the time point marked by arrow. The value of Cell Index at this time was set to “1,” and all subsequently recorded values of Cell Index were then normalized to this point. The decrease in cell index shows the disruption of the HUVEC monolayer by the invading GBM cells. Representative data from three independent experiments performed for each pdGSC. GBM, glioblastoma; CIM, cell invasion migration; HUVEC, human umbilical vein endothelial cells; pdGSC, patient-derived GBM stem cell; cDNA, complementary DNA.

In this study, we investigated whether manipulating CD97 expression levels affected the invasion of pdGSCs *in vivo* and compared results with *in vitro* studies. First, endogenous CD97 levels were assessed in multiple cell lines and three primary pdGSCs from our collection were selected: GBM1, GBM12, and GBM22 (Fig. 1*A*). Then, lentivirus-delivered shRNA was used to knockdown CD97 expression (shCD97), and CD97-encoding complementary DNA (cDNA) was used to overexpress this receptor (CD97-OE) in pdGSCs. In CD97-OE, the N terminus was tagged with FLAG, in order to detect exogenously expressed CD97. GFP-transduced cells were used as the controls (WT).

Western blot analysis with anti-CD97 antibody demonstrated that two out of the three shRNAs tested effectively reduced the expression of the receptor (Fig. 1*B*). The shRNA-2 was found to have the highest knockdown effect, reducing expression by approximately 80%, and was therefore used in all subsequent experiments. The changes in CD97 expression in pdGSCs after lentivirus transduction were confirmed by immunostaining with CD97 antibody. The GFP fluorescence and FLAG immunostaining were used to demonstrate high transduction efficiency, with almost all cells being positive for these markers (Fig. 1*C*). The specificity of CD97 antibodies in cells was verified by immunostaining of nonmodified GBM cells and HEK293 cells used as a negative control. As expected for mature receptors, CD97 immunoreactivity colocalized with fluorescently conjugated wheat germ agglutinin lectin, a marker of the plasma membrane in GBM22 and U87 cells, with low nonspecific background in HEK293 cells (Fig. S1*A*).

### CD97 knockdown increases invasiveness of stably transduced pdGSCs *in vitro*

Previous studies using U87 and U251 GBM cell lines (19) as well as our earlier data using primary pdGSCs (35) have shown that knockdown of CD97 decreased GBM cell invasion *in vitro*. In the cell lines (19), CD97 was silenced by transient transfection and analyzed after 48 h. In our previous experiments, the primary pdGSCs were transduced with lentivirus carrying shRNA and plated the next day, prior antibiotic selection (35). In the current study, to achieve a near 100% transduction efficiency of the constructs, the transduced pdGSCs were selected with puromycin for at least 5 days (Fig. 1*C*). The antibiotic selection was also necessary for the subsequent studies of the pdGSCs xenografts (Figure 4, Figure 5, Figure 6, Figure 7). The cells were seeded in a two chamber cell invasion migration (CIM) plate coated with Matrigel and their invasion toward 10% fetal bovine serum (FBS) was monitored for 36 h using real-time cell analysis (RTCA) (Fig. 1*D*). We found that the pdGSCs stably transduced with shCD97 exhibited increased invasiveness, surpassing those overexpressing CD97 and WT controls. Such an effect of CD97 knockdown was observed in all three GBM1, GBM12, and GBM22 primary cells (Fig. 1*D*). The increase in invasiveness upon CD97 knockdown contrasted with the previous findings on GBM cell lines and nonselected pdGSCs (19, 35). We attribute this controversy to the long-term effect of pdGSC culture under the pressure of puromycin selection. One obvious effect is that only cells harboring the shCD97 construct survive. Additionally, we hypothesize that the substantial reduction in CD97 levels leads to adaptive changes in some or all of the surviving pdGSCs, resulting in the acquisition of a more invasive phenotype.

The Matrigel used in the two-chamber plate assay mimics the ECM, which acts as a barrier to cell movement. Cancer cells usually overcome this barrier by releasing enzymes that degrade the ECM (36). However, *in vivo*, tumor cells need to disrupt both ECM and cell-to-cell interactions to invade tightly connected tissue. To investigate the impact of CD97 expression on the ability of GBM cells to disrupt cell-to-cell interactions, we employed an alternative assay based on a different principle (37). In this assay, a lawn of human umbilical vein endothelial cells (HUVECs) was formed on the surface of an RTCA E-plate. Cultured as a monolayer, these cells adhere to each other *via* adherens junctions (AJs) mediated by vascular endothelial (VE)-cadherin interactions (38). If cocultured tumor cells create an environment that disrupts these junctions, the impedance decreases due to reduced surface occupied by HUVECs. This may indicate that such tumor cells are more conducive to invasiveness. The results showed that the CD97 knockdown stimulated this behavior in two of the three pdGSCs (Fig. 1*E*). The effects of the changes in CD97 expression were not as pronounced as in the experiment with the Matrigel (Fig. 1*D*), and it occurred in two out of three pdGSCs. Except for the data with the single primary GBM cell line, two independent *in vitro* assays support the notion that continuous suppression of endogenous CD97 levels may increase pdGSCs invasion.

### CD97 influences HUVECs cell-to-cell adhesion by the secretion of soluble factors

**Figure 2:**
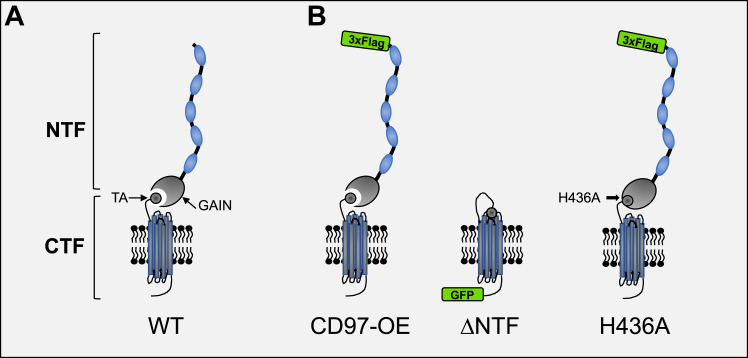
**Schematic domain structure of CD97 and its mutants.***A*, The WT CD97 receptor (WT) consists of a C-terminal fragment (CTF) and large extracellular N-terminal fragment (NTF). The CTF includes a 7-TM GPCR domain and a tethered agonist (TA). The NTF contains 3 to 5 epidermal growth factor domains (*blue ellipses*) and the GPCR autoproteolysis–inducing (GAIN) domain that is noncovalently bound to TA. The GAIN domain prevents TA from activating the receptor. The complete dissociation of NTF releases the inhibition, causing irreversible activation of CD97. *B*, three constructs were created to investigate the function of CD97. In CD97-OE construct (NCBI, P48960), three FLAG tag copies were cloned in-frame within the N terminus of NTF fragment. Truncation of NTF at the autocleavage site created the ΔNTF mutant of CD97 that constitutively activates Gα_12/13_. For detection in cells, the C terminus of this mutant was fused in-frame to GFP. The H436A mutant has a His-to-Ala point mutation in the GAIN domain to disable self-cleavage. The permanent fusion of the NTF to the CTF prevents TA from activating downstream Gα_12/13_ signaling. Like the CD97-OE construct, the noncleavable H436A mutant is tagged with 3xFLAG for immunodetection in cells.

The *in vitro* invasion assay using HUVECs suggested that GBM cells may impact the disruption of HUVEC monolayers through the release of certain cytokines (39, 40). We reasoned that this process may be regulated not only by CD97 levels but also by the activation state of the receptor. To test this hypothesis, two functional mutants of CD97 were created: a constitutively active mutant lacking the extracellular domain (ΔNTF) and a dominant-negative mutant with the H436A amino acid substitution that prevents autocleavage at the GPS site (Fig. 2). These mutants activate and inhibit downstream Gα_12/13_-mediated signaling, respectively (17, 22). The ability of the GFP-fusion ΔNTF mutant to activate Gα_12/13_ was confirmed using a reporter assay that responds to activation of the Rho pathway (41) (see Fig. S2). The two mutant constructs were cloned into lentiviruses and stably transduced into pdGSCs.

**Figure 3:**
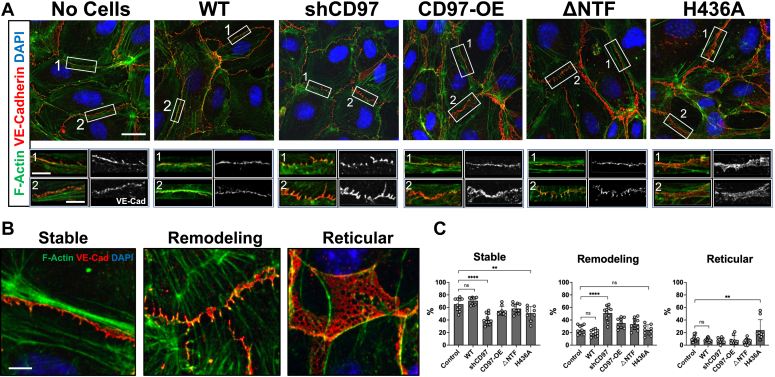
**CD97-modified pdGCS affect the integrity of endothelial cell–cell adherens junctions by secretion of soluble factors.***A*, representative images of HUVECs exposed to the media collected from either control GBM22 cells (WT) or cells modified by CD97 constructs. Scale bar represents 20 μm upper panels, 5 μm magnified insets. HUVECs treated by GBM growth media that was not exposed to cells were used as a control (no cells). The two insets under each image show the close-up images of two representative adherens junctions (AJs) denoted by *white boxes*. Cells were stained with antibodies against AJ marker VE-cadherin (*red*), phalloidin to identify filamentous actin (F-actin, *green*), and DAPI to label nuclei (*blue*). *B*, representative images show the examples of morphological characteristics used to identify a particular type of AJ: stable mature junctions where VE-cadherin overlaps with faint cortex F-actin and is aligned by thick parallel actin bundles; remodeling junctions, in which perpendicular oriented VE-cadherin colocalizes with radial actin bundles; and reticular junctions characterized by a 3-dimensional VE-cadherin network and has low level of actin filaments. Scale bar represents 5 μm. *C*, calculated percentages of different AJ types in HUVECs after pdGSC’s conditional media exposure. Data derived from three independent experiments where 3 to 4 visual fields per condition were analyzed and each dot on the graph represents the average of 30 to 60 AJs per field. The values are mean ± SD (N varies between 10 and 12). Two-way ANOVA Tukey’s multiple comparison test was performed; not significant (ns) *p* > 0.1, ∗∗*p* < 0.01, ∗∗∗∗*p* < 0.0001. GBM, glioblastoma; HUVEC, human umbilical vein endothelial cells; pdGSC, patient-derived GBM stem cell; VE, vascular endothelial.

We collected conditioned media from GBM22 cells modified with WT, shCD97, CD97-OE, ΔNTF, and H436A constructs and applied it to HUVECs monolayers. After the exposure to the media, the HUVECs were costained with antibodies against VE-cadherin, a marker of cell-cell AJs, and phalloidin to visualize filamentous actin (F-Actin) (Fig. 3*A*). As a control, HUVECs were also treated with GBM growth media not exposed to cells.

The dynamic nature of cell-cell AJs makes it difficult to draw conclusions based on a single time point snapshot (38). Therefore, to analyze the static confocal images, we assessed the number and representation of morphologically distinct types of junctions. Three AJ types were identified: stable mature, active remodeling, and reticular junctions (Fig. 3*B*). The active remodeling and reticular junctions have been found to be more permeable to cancer cells than stable mature junctions (38, 42, 43). Therefore, an increase in the proportion of either of these two AJ types may suggest that GBM cells release soluble factors that enhance the permeability of the endothelial cell barrier.

To assess the prevalence of specific AJ types, we examined the staining patterns of VE-cadherin and F-Actin within the HUVECs junctions. Figure 3*B* exemplifies the morphological characteristics used to identify the specific junction types. In stable mature AJs, VE-cadherin overlaps with faint cortex F-actin and is aligned by thick parallel actin bundles; remodeling junctions are characterized by perpendicular-oriented VE-cadherin staining colocalizing with radial actin bundles; reticular AJs exhibit three-dimensional VE-cadherin network that has low level of actin filaments.

Guided by these morphological characteristics, we analyzed high magnification confocal images to calculate the prevalence of each AJ type between adherent HUVECs. These quantification results are presented in Figure 3*C*. There was no significant difference between the impact of the media collected from the WT pdGSCs *versus* untreated media (control). However, media collected from pdGSCs with CD97 knockdown produced a significant increase in the number of actively remodeling AJs, while the media from H436A-modified cells increased the proportion of reticular AJs. Both active remodeling and reticular types of AJs favor cancer cell permeability (38, 42, 43). Therefore, our results suggest that the knockdown of CD97 or inhibition of CD97 downstream activity (*i.e.*, H436A) in pdGSCs can promote the secretion of chemokines or other soluble factors disrupting cell-cell adhesion, thus potentially creating an invasion-favorable tumor environment.

Next, we investigated whether CD97 influences pdGSCs behavior *in vivo*.

### Effect of CD97 on GBM xenograft size and cell proliferation *in vivo*

**Figure 4:**
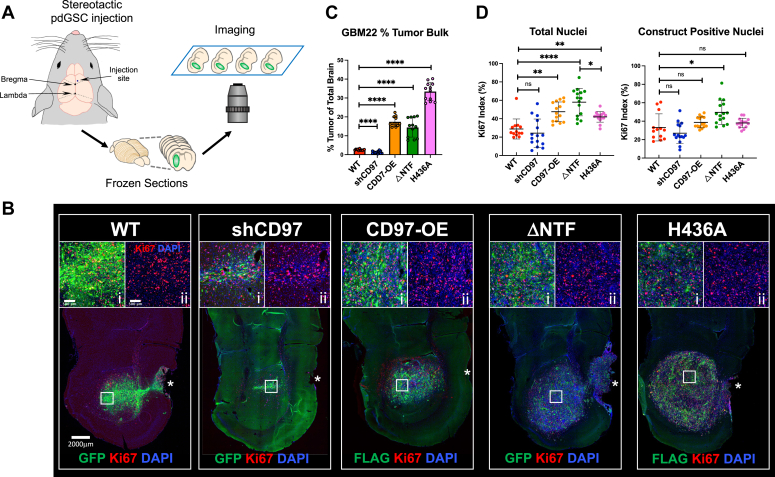
**Effect of CD97 activity on GBM cell proliferation in mouse brain tumors.***A*, schematic workflow to study modified pdGSCs *in vivo*. Mice were stereotactically injected in the right frontal cortex anterior from bregma. After tumor development, brains were perfused, fixed, and harvested. Frozen sections were prepared, stained, and imaged as described in Experimental procedures. *B*, representative images of the brain sections with tumors formed by GBM22 cells expressing each construct (scale bar represents 2000 μm). Tumor cells were identified by either GFP fluorescence (WT, shCD97, and ΔNTF) or staining with anti-FLAG antibody (CD97-OE and H436A) (*green*). All sections were stained with the Ki67 antibody (*red*) and DAPI (*blue*). The insets (scale bar represents 500 μm) at the top of each image show close-up images of representative regions of interest (ROI, *white square*). The left insets (“i”) show the merge images of all three channels demonstrating the expression of CD97 construct in each tumor. The right insets (“ii”) show only Ki67 and DAPI staining used for calculations in (*D*). *Asterisks* indicate the location of the injection site. *C*, the graph displays percentage of the brain slice area occupied by the tumor. The estimated measurements were obtained from 5 to 6 brain sections located near the injection site and covered approximately 0.5 mm of brain tissue thickness. One-way ANOVA with Dunnett’s T3 multiple comparison test, n.s. = *p* > 0.1, ∗∗∗∗*p* < 0.0001; N = 3 mouse brains (5 brain slices per condition for each mouse), N = 12, mean ± SD. *D*, quantification of tumor proliferation rate using the Ki67 index calculated as a ratio of Ki67-positive to total DAPI-positive nuclei (see text for details). Calculations of Ki67 index were done for each ROI area outlined by *white square* in (*B*) as an example. The “Total Nuclei” graph shows the Ki67 index calculated for all cells (with inclusion of host cells) in the ROI area. The “construct-positive nuclei” graph displays the Ki67 index calculations only for GFP- or Flag-positive cells within the ROI. One-way ANOVA with Dunnett’s T3 multiple comparison test, n.s. *p* > 0.1, ∗*p* < 0.1, ∗∗*p* < 0.01, ∗∗∗∗*p* < 0.0001; N = 15 (3 mouse brains x five brain slices per condition for each mouse), mean ± SD. GBM, glioblastoma; pdGSC, patient-derived GBM stem cell; ROI, region of interest.

We utilized a mouse xenograft model by stereotactically implanting modified pdGSCs into the right frontal lobe of the brain (Fig. 4*A*). Two primary pdGSCs, GBM22 and GBM1, were selected for the *in vivo* experiments (Fig. 1). Based on our previous findings, the average survival time for mice implanted with either of these primary cells was approximately 8 weeks. To ensure comparability of the tumor xenografts, we harvested the brains 6 weeks post implantation. Frozen sections of each brain were prepared and imaged using a high-resolution slide scanner microscope (Fig. 4*A*). Here, we present and discuss the results for GBM22, while the data for GBM1 can be found in Supplemental Figures (Figs. S3, S5, S6 and S8).

As in our *in vitro* experiments, to enable detection of tumor cells within the tissue, the cells transduced with WT and shCD97 constructs also expressed GFP, while ΔNTF had GFP cloned in-frame with an expressed mutant protein (see Experimental procedures and Fig. 2). The CD97-OE and H436A mutant had a FLAG tag attached to the N terminus of CD97 (Fig. 2*B*). The tag introduced at this location was proved to not interfere with CD97 function (44). Before the surgery, the transduced cells underwent stringent puromycin selection to ensure that most cells expressed the desired construct (Fig. 1*C*, for cell preparation, see Experimental procedures).

**Figure 5:**
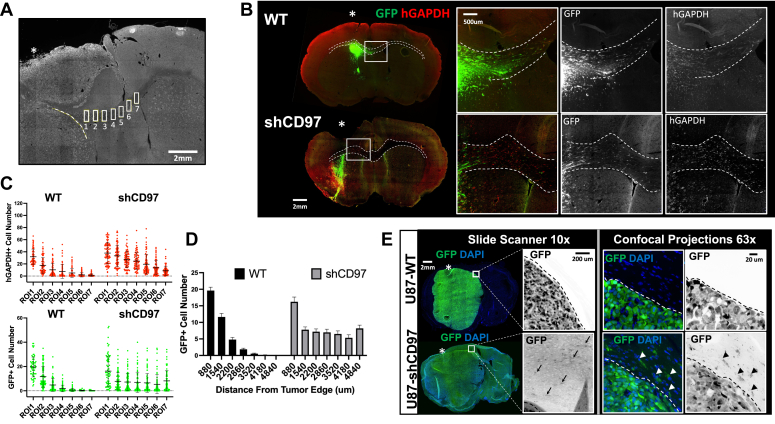
**CD97 knockdown does not inhibit migration of primary pdGSC along Corpus Callosum.***A*, representative brain slice stained with hGAPDH antibody illustrates the position of seven ROIs along CC. The dash line marks the tumor border, and the asterisk marks the position of the injection site. The number of GBM cells within the ROIs is determined and used for measuring cell migration along the CC; see details in the Experimental procedures. *B*, representative images of the brain slices with tumors formed by control (WT) and knockdown (shCD97) GBM22 cells (scale bars represent 2000 μm). The tumor cells express GFP (*green*), and the brain slices were stained with hGAPDH antibody (*red*) to label all human cells. CC is outlined with the dash line, and the asterisk marks the injection site. The insets to the *right* show the close-up merged and single channel images (scale bar represents 500 μm) of the outlined areas (*white squares*). *C*, the graphs show the numbers of hGAPDH-positive (*red*) and GFP-positive (*green*) cells within ROI (as shown in (*A*)) along CC. The data was obtained by analyzing 25 brain sections for each of the three mice per condition. The total of 75 data points were plotted for each ROI. N = 75, mean ± SD. *D*, to better illustrate the migration of cells expressing the constructs, the graph shows the calculated average number of GFP-positive cells within all ROIs at the corresponding location along CC in each brain slice. N = 75, mean ± SEM. *E*, the representative images showing the sections of mouse brain with tumors formed by U87 cells transduced with GFP (U87-WT) or shCD97 (U87-shCD97) constructs displaying GFP fluorescence (*green*) and stained with DAPI (*blue*). *Left* panels show low magnification images of the brain slices taken by the Slide Scanner automated microscope (scale bar represents 2000 μm). The *asterisks* mark the injection site. GFP single channel images to the right show the enlarged images of the outlined square areas at the edge of the tumors (scale bar represents 200 μm). *Arrows* point to the GFP-positive “streaks” which only can be observed in the brains with shCD97 tumors. The high magnification confocal images shown in the right panels (scale bar represents 20 μm) illustrate the presence of elongated GFP-positive U87 cells with CD97 knockdown (*arrowheads*) migrating along the tumor border (*dashed lines*) in the brain parenchyma. No invading GFP-positive U87 cells were detected in control tumors. CC, corpus callosum; GBM, glioblastoma; hGAPDH, human GAPDH; pdGSC, patient-derived GBM stem cell; ROI, region of interest.

**Figure 6:**
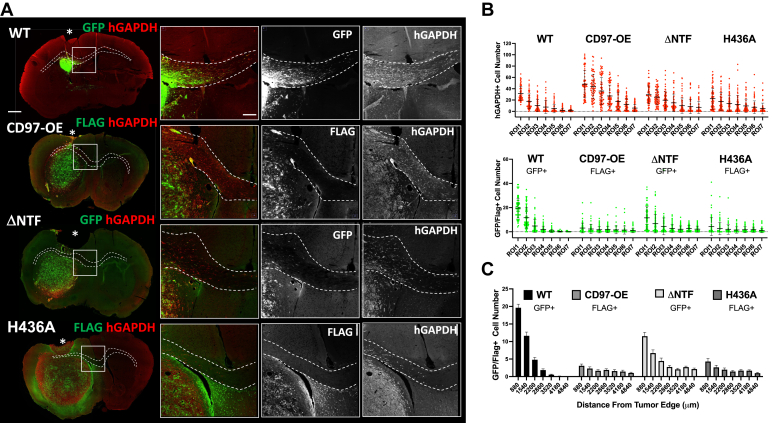
**Effect of CD97 overexpression and its activity on pdGSCs migration along Corpus Callosum of mouse brains.***A*, the representative images of the brain slices with tumors formed by GBM22 cells expressing control (WT), FLAG-tagged CD97 (CD97-OE), constitutively active ΔNTF mutant fused to GFP (ΔNTF), and FLAG-tagged noncleavable CD97 mutant (H436A). All brain sections were stained with hGAPDH antibody (*red*) to label human cells. Cells expressing WT and ΔNTF were identified by GFP fluorescence, while FLAG antibody was used to label NTF in CD97-OE– and H436A-expressing cells (*green*). (Scale bar represents 2000 μm). Outlined boxed areas are enlarged and displayed in the images to the *right* showing merged and single channel staining (scale bar represents 500 μm). The *asterisk* marks the injection site, and the *dashed lines* mark position of Corpus Callosum. *B* and *C*, the quantification of modified pdGSCs migrating along CC (marked by *dash lines* in *A*) was performed as described for Fig. 5, *A* and *B*. Like in Fig. 5, *C* and *D*, graphs show the calculated raw numbers of migrating hGAPDH-positive cells (*red*) and GFP- or FLAG-positive (*green*) cells in each ROI, mean ± SD (B), and the average number of GFP- or FLAG-positive cells per ROI, mean ± SEM (*C*). CC, corpus callosum; GBM, glioblastoma; hGAPDH, human GAPDH; NTF, N-terminal fragment; pdGSC, patient-derived GBM stem cell; ROI, region of interest.

**Figure 7:**
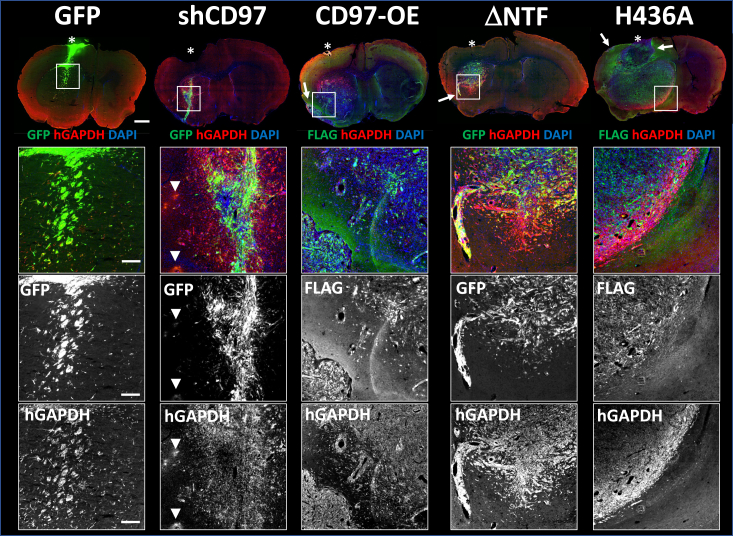
**The distinct pattern of pdGSCs invasion from the edge of CD97-modified tumors.** Representative brain slices from GBM22 tumors (scale bar represents 2000 μm) generated by control (WT) or modified pdGSCs stained with hGAPDH antibodies and GFP or anti-FLAG immunofluorescence used to identify cells expressing the corresponding construct as in Figures 5 and 6. *Asterisk* label estimated injection site. The boxed areas in each brain section were magnified to provide a closer view of the tumor cells infiltrating the brain tissue. Merged as well as single channel images are used to visualize the distinct patterns of individual cell infiltration (scale bar represents 500 μm). The CD97 knockdown cells (shCD97) display an invasive phenotype characterized by elongated and convoluted cell bodies. Notably, at the outer edges of these tumors, we observed patchy fluorescence in both channels resembling the staining of cell remnants (*white arrowheads*). The ΔNTF, CD97-OE, and H436A tumors are larger and have finger-like projections extending into the brain (*white arrows*). The FLAG-positive cells within CD97-OE and H436A tumors (*green*) are primarily located within the central core, indicating the presence of NTF. CD97-OE tumors exhibit a significant presence of hGAPDH-positive cells that lack FLAG fluorescence, infiltrating brain parenchyma that is not observed in H436A tumors. The ΔNTF tumors display extensive brain infiltration of double-stained cells at the tumor border. GBM, glioblastoma; hGAPDH, human GAPDH; NTF, N-terminal fragment; pdGSC, patient-derived GBM stem cell.

To facilitate quantitative analysis of the tumors, it was necessary to define the border between the tumor bulk and surrounding brain tissue. This allowed us to estimate the overall tumor size (Fig. 4*C*) and also investigate the invasion of single GBM cells from the tumor (Figure 5, Figure 6, Figure 7). To detect all the transplanted xenografts, the mouse brain slices were stained with antibodies recognizing only human GAPDH (hGAPDH). Two other markers were used to label specific CD97 constructs (Figs. 2, 4 and 5 and S5). The cells were costained with anti-FLAG antibodies to detect the FLAG-tagged NTF in CD97-OE– and H436A-expressing tumors (Fig. 2). The control WT cells, cells expressing shCD97, and ΔNTF cells were visualized by GFP fluorescence.

The visual assessment of the tumors formed by each construct revealed high variation in tumor morphology and the degree of their infiltration into the brain tissue. The most uniform tumor shapes were observed near the injection site, which was identified by the scar left by the injection needle. Therefore, these regions were used as reference points to estimate the sizes of the tumor bulk (see Experimental procedures for more details). The measurements showed that the xenografts derived from pdGSCs with CD97 knockdown were significantly smaller than the WT control, for both GBM22 and GBM1 tumors. On the other hand, cells overexpressing CD97 or its mutants produced significantly larger tumors (Figs. 4*C* and S3*A*).

To investigate whether the changes in tumor size were caused by differences in tumor cell proliferation rates, the brain sections were costained with the proliferation marker Ki67 and DAPI. The pdGSCs expressing the constructs were identified by GFP fluorescence or FLAG immunostaining (Fig. 4*B*, insets). The tumor proliferation rates were calculated as the ratio of Ki67-positive nuclei to the total number of nuclei identified by the DAPI staining. The values of Ki67 index were expressed in percentages. For these calculations in each brain section, we selected a region of interest (ROI) within the tumor bulk located close to the tumor center but avoiding the necrotic tissue (Fig. 4*B*).

The cellular composition of GBM tumors may include proliferating host cells such as microglia, astrocytes, vascular, and other cells (20, 45); therefore, two approaches were used to measure the Ki67 index. First, we calculated the percentage of Ki67-positive cells among all cells included in the ROI (Fig. 4*D*, left graph). The Ki67 index obtained this way factors in the contribution of proliferating host cells. In the second approach, we identified the percentage of Ki67-positive nuclei in only those cells that possessed CD97 constructs. The results obtained by this approach would determine whether CD97 expression levels or its activity directly affected proliferation of the tumor cells. (Fig. 4*D*, right graph). To do this, in ImageJ, the mask of the green fluorescence was applied to the Ki67 and DAPI channels to exclude any GFP- or FLAG-negative cells (see “Experimental procedures”). The close-up images of the ROIs in Figure 4*B* illustrate the merged Ki67 and DAPI staining with (inset “i”) or without (inset “ii”) GFP or FLAG fluorescence.

For GBM22 tumors, the Ki67 index calculated by the first method, which includes all cells in the ROI, showed some correlation between the apparent tumor size and proliferation (Fig. 4, *C* and *D*, left graph). For example, the smallest tumors formed by cells with CD97 knockdown had a reduced Ki67 index compared to control tumors, although the difference was not significant (24 ± 15.3% *vs*. 29 ± 10.9%, respectively). Tumors overexpressing CD97 or its mutants had the largest tumors and significantly increased proliferation compared to tumors formed by WT cells. However, other result details did not support the correlation between proliferation and the tumor size. For example, the most significant increase in Ki67 index (58 ± 14.9%) was observed in tumors expressing the constitutively active ΔNTF mutant. Yet, these tumors were approximately half the size of those generated by the dominant-negative H436A mutant, for which the Ki67 index was significantly lower (42 ± 5.7%) (Fig. 4, *C* and *D*). Moreover, ΔNTF tumors were comparable in size with tumors overexpressing CD97 (CD97-OE), for which Ki67 index was also lower though the difference was not significant (48 ± 10.6%).

The GBM1 tumors showed similar trends for the sizes of tumor bulk and Ki67 index, although the overall proliferation rate of GBM1 tumors was much higher than tumors generated by GBM22 cells (compare Figs. 4, *C* and *D* and S3). It was also observed that GBM1 tumors derived from the CD97 knockdown cells were even smaller in size than the knockdown tumors generated by GBM22 cells.

The second approach to measure Ki67 index, which included only the construct-positive tumor cells, showed that significant increase in tumor cells proliferation only occurred with the constitutively active ΔNTF mutant. The Ki67 index of tumor cells expressing other constructs did not show a significant difference compared to control (Figs. 4*D* and S3*B*, right graphs). Taken together, these data suggest that CD97 expression levels have no effect on tumor cells proliferation *in vivo*. However, activation of CD97 downstream signaling can affect tumor cell proliferation.

### Invasion of CD97-modified GBM cells *in vivo*

GBM tumors are known to be highly invasive within the brain, but they rarely metastasize outside of the central nervous system. The invasion of the tumors commonly occurs along the white matter tracts and blood vessels with individual tumor cells also disseminating into the brain. Our objectives were to examine the CD97-dependent migration of modified pdGSCs along the corpus callosum (CC) and to observe their infiltration into the brain tissue of tumor parenchyma.

To achieve these goals, we began by defining the tumor border to differentiate between tumor bulk mass and individual cells or very small cell clusters migrating away from the tumor which we refer to as single cell migration. The tumor borders were defined using the same method as described above for the assessment of tumor bulk (Fig. 4, also see Experimental procedures). The xenografts formed around the injection site were suitable for examining tumor cell migration along white matter tracts as they crossed the myelinated fibers of CC. The border of the tumor bulk was chosen to be a starting point to measure the distances traveled by individual cells, as the growing tumors often cover or compress the CC fiber tracts near the injection sites. We then calculated the number of the migrating pdGSCs within seven equally spaced ROIs along the CC (Figs. 5*A* and S4). This approach enabled us to cover a total distance of approximately 5000 μm traveled by migrating tumor cells. Furthermore, by placing corresponding ROIs in each consecutive brain section of the stack (Fig. 4*A*), we could encompass up to 500 μm of the CC thickness.

As described above, hGAPDH immunostaining was used to identify all pdGSCs, GFP fluorescence to detect tumor cells expressing the corresponding constructs, and FLAG immunostaining to label FLAG-tagged NTF in CD97-OE and H436A constructs (see Fig. 2). The red channel was assigned to hGAPDH and the green channel to GFP or FLAG fluorescence (Figs. 5, *B* and *C*, S5 and S6). It is important to emphasize here that *in vivo*, the NTF may irreversibly dissociate from the GPCR domain. As soluble ligand, the NTF can interact with other binding partners such as cell surface receptors or ECM (24, 25). After NTF dissociation, the invading tumor cells expressing constructs with FLAG-tagged NTF (Fig. 2*B*) would still be positive for hGAPDH but negative for FLAG immunofluorescence. Furthermore, FLAG-tagged NTF (often called “soluble CD97”) could also be detected in regions of the mouse brain tissue devoid of the human cells. It is possible that a small percentage of injected pdGSCs may not harbor any construct and therefore lack green fluorescence. However, stringent antibiotic selection before implantation makes this possibility highly unlikely.

### The effect of CD97 knockdown on GBM cell invasion *in vivo*

To examine the impact of reduced CD97 expression on GBM invasion, we compared brain section images for GBM22 and GBM1 pdGSCs transduced with control lentivirus (WT) and CD97-targeting shRNA (shCD97) (Figs. 5, S5 and S6). Both constructs expressed GFP. Image analysis detected hGAPDH- and GFP-positive cells migrating along myelinated fibers of CC for both WT and shCD97 conditions (Figs. 5, *B* and *C*, S5 and S6*A*). The numbers for detected GFP-positive cells were smaller than the numbers of hGAPDH-positive cells (compare Y axes for green and red data points in Fig. 5*C*). This difference in numbers of the migrating cells could be explained by individual cell variability in the GFP fluorescence intensity as compared to hGAPDH staining and as the result, missing some GFP-positive cells with GFP fluorescence under the threshold. Importantly, the CD97 knockdown cells were detected in CC much further from the tumor edge compared to control WT cells (Fig. 5*D*). These results correlated with our *in vitro* invasion assays (Fig. 1*D*) and demonstrated that *in vivo*, primary pdGSCs with reduced levels of CD97 still maintain their invasive properties.

To further investigate the impact of CD97 knockdown on GBM invasion, we asked if the reduced CD97 expression may affect the invasiveness of U87 tumors. U87 GBM cells express high levels of CD97 (Figs. 1*A* and S1) and are known to produce fast-growing noninvasive tumors with very defined borders (11). U87 cells were transduced with either GFP or shCD97 constructs and after puromycin selection, implanted into mouse brains. Immunostaining with CD97 antibodies confirmed significant reduction in CD97 expression in tumors after knockdown (Fig. S1*B*). Analysis of the generated tumors at low magnification revealed large compact tumors with sharp borders for both the control and CD97 knockdown constructs (Fig. 5*E* Left Panels, Fig. S7). This suggested that CD97 knockdown did not result in massive tumor invasion. However, upon closer examination, we observed GFP-positive "streaks" in the brains of U87 knockdown tumors, which were not present in the control tumors (Figs. 5*E* and S7, arrows). We hypothesized that these GFP-positive "streaks" are to be elongated invading U87 tumor cells. Subsequent analysis of images taken at high magnification using a confocal microscope supported this interpretation: it showed the CD97 knockdown GFP-positive U87 cells outside the tumor border (Fig. 5*E*, arrowheads). These infiltrating cells exhibited elongated cell bodies that are characteristic of invading cells. In contrast, no GFP-positive cells were detected in proximity to the control WT U87 tumors. These experiments demonstrate that reducing CD97 expression promotes an invasive phenotype of U87 tumors, albeit in a small subpopulation of cells.

### CD97 activity and GBM cell invasion *in vivo*

Next, we explored the overexpression of CD97 and the influence of its functional activity on the invasiveness of GBM xenografts. Figure 6*A* shows representative images of the GBM tumors derived from GBM22 pdGSCs transduced with WT, CD97-OE, ΔNTF, and H436A constructs. Quantitative image analysis of all examined tumors detected hGAPDH-positive cells up to 5 mm along CC from the tumor edge (Fig. 6*B*, upper graph). Similar to the CD97 knockdown (Fig. 5), the analysis showed that the number of GFP- or Flag-positive cells was lower in all tumors than the numbers of hGAPDH-positive cells (Fig. 6*B*, note the difference in Y-axis scale between two graphs).

The examination of FLAG-positive cell migration along CC fibers revealed that CD97-OE and H436A constructs had significantly fewer migrating cells than the WT and ΔNTF constructs (Fig. 6*B*, lower graphs). The average number of FLAG-positive pdGSCs for CD97-OE and H436A per ROI was limited to only 2 to 3 single cells, even near the tumor border (Fig. 6*C*). Such a reduction was particularly notable in GBM1 pdGSCs (Figs. S5 and S6*B*). At the same time, we detected high numbers of hGAPDH-positive cells for CD97-OE (up to 100 cells per ROI) and for H436A (up to 60 cells per ROI) constructs (Figs. 6*B* and S6, upper graphs). Assuming that all hGAPDH-positive cells harbor the transduced construct, the data suggest that GBM cells can only migrate along CC when their extracellular NTFs dissociate from the GPCR moiety. Interestingly, for H436A tumors, a significant number of hGAPDH-positive but FLAG-negative cells migrating along CC was observed (Figs. 6*B* and S6, upper graphs). The H436A amino acid substitution should prevent the autoproteolysis of the NTF (22), but it is possible that *in vivo*, H436A mutant is cleaved by extracellular proteases liberating the FLAG-tagged NTF. Consistent with the idea that soluble CD97 can diffuse through the tissue and act as a ligand for other targets, we observed the FLAG staining in hGAPDH-negative cells outside CC (Fig. 6*A*, bottom row).

To determine whether CD97 influences GBM cell invasion into the brain parenchyma, we examined modified pdGSCs infiltration into the brain tissue around tumor bulk. The irregularity of tumor morphology precluded quantitative measurements limiting our analyses to qualitative assessments. Figure 7 shows representative brain slices with tumors formed by GBM22 cells expressing different constructs. The enlarged images of the boxed areas demonstrated that tumor cells infiltrated the brain tissue in all tumors analyzed. However, there were some noticeable differences. For instance, in the small tumors with CD97 knockdown, the center was filled with GFP and hGAPDH double-positive cells exhibiting an elongated convoluted morphology, characteristic of an invasive phenotype. Numerous single cell migration away from the tumor bulk was also detected. Yet, the tumor periphery was surrounded by diffused and patchy hGAPDH and GFP staining resembling cell remnants (Fig. 7, shCD97, arrowheads in the insets). These observations suggest that CD97 knockdown can cause cell death in some subpopulations of GBM tumor cells while sparing other cells and even making them more invasive. The reduced viability of tumor cells expressing shCD97 may also explain significant decrease in these tumors size (Figs. 4*C*, and S3*A*).

The morphology of the ΔNTF pdGSCs at the tumor edge and their extensive single cell migration from the tumor mass closely resembled that of the shCD97 tumors. However, the hGAPDH staining of ΔNTF tumor cells clearly reflected the characteristics of live cells (compare panels for shCD97 and ΔNTF in Fig. 7). Another notable difference between CD97 knockdown and deletion of NTF is that the ΔNTF-expressing cells formed larger size xenografts than the knockdown tumors. This suggests that constitutively active CD97 does not impair tumor cell viability.

The H436A mutant pdGSCs produced massive tumors sending long protrusions, which appeared to be a continuation of the tumor bulk or collective cell migration (Fig. 7, H436A, arrows). These protrusions squeeze through the brain tissue as a group of densely packed cells rather than individually infiltrating pdGSCs. Despite the very large tumor size, only a few hGAPDH- and FLAG-positive cells were detected far from the tumor edge. These observations suggest that the invasion mechanism employed by H436A mutant tumors is different from the tumors expressing other constructs.

The tumors formed by the GBM cells overexpressing the nonmodified CD97 receptor (CD97-OE) also displayed large protrusions (Fig. 7, CD-OE, arrow). Similar to the H436A tumors, only a few FLAG-positive cells were detected far from the tumor border. However, the hGAPDH-positive cells that lost the FLAG-tagged NTF showed a significant single cell infiltration in the brain tissue.

## Discussion

In this study, we investigated the functional role of CD97 in GBM. This aGPCR is involved in cell migration and invasion in various metastatic cancers (39, 46, 47, 48, 49). Association of high levels of CD97 with metastasis suggests that decreasing its expression may reduce cell motility and invasion. Indeed, many studies have found that CD97 knockdown inhibits invasion and migration in various primary cancer cells and cell lines (15, 17, 19, 50, 51). However, in fibrosarcoma cells, migration was also inhibited by the overexpression of CD97 (28). A recent review concluded that influence of CD97 on proliferation, adhesion, and/or invasion depends on the cell type and specific tumor microenvironment (52). Here, we investigated CD97 function in human pdGSCs and for the first time, analyzed the impact of CD97 knockdown, overexpression, and functional mutations in mouse brain xenografts.

### CD97 in tumor growth and proliferation

Previous studies attempted to determine whether CD97 plays an active role in GBM growth. Initially, it was found that CD97 knockdown in U87 GBM cells had no effect on proliferation *in vitro* (19). A more recent study investigated association between CD97 levels in neoplastic cells and proliferation pathways using transcriptomic analysis of human GBM specimens but did not arrive to clear conclusion (20). Our current study addressed the cause-and-effect relationship between CD97 activity and tumor progression by manipulating the CD97 levels and changing its activity with functional mutations.

Neither knockdown of CD97 nor its overexpression significantly changed the Ki67 index of the tumor’s xenografts. However, the knockdown led to a dramatic reduction of tumor mass, while overexpression caused tumor enlargement (Fig. 3*C*). These results suggest that the significant reduction in tumor volume in the knockdown may be caused by the decreased survival of the implanted cells. Indeed, we noticed the distinct pattern of hGAPDH staining of the shCD97 tumors resembling cell debris rather than the shape of healthy cells. In contrast, the tumor enlargement caused by CD97 overexpression suggested the increased cell viability. In fact, previous findings showed that CD97 mediates resistance to extrinsic apoptosis by promoting cell adhesion *via* NTF interaction with integrins (53). This hypothesis is supported by the previous analysis of the public genomic database that revealed inverse correlation between higher levels of CD97 and GBM patient’s survival (19). Therefore, our results obtained with GBM xenografts are consistent with these data.

Our analysis of CD97’s functional mutants provided additional mechanistic insights into whether this receptor can influence the proliferation of GBM cells and tumor growth. The results showed that expression of the CD97 ΔNTF mutant increases Ki67 proliferation index, indicating that constitutive activity of CD97 promotes proliferation (Fig. 3, *A* and *B*). This effect is consistent with the currently accepted model of CD97 activation where dissociation of NTF leads to stimulation of the downstream Gα_12/13_-dependent progrowth Rho pathway (17, 22, 27, 54). Interestingly, despite the significant increase in proliferation (*i.e.*, Ki67 index), there was no corresponding increase in the size of the ΔNTF tumors compared to those with the overexpression of the WT CD97 (CD97-OE). It is known that continuous GPCR activation could lead to desensitization, internalization, and subsequent degradation of the receptor (55). Thus, the ΔNTF-expressing tumors may have lower amount of the endogenous CD97 receptor on the cell surface, partially recapitulating the effect of CD97 knockdown.

Overexpression of the noncleavable H436A mutant resulted in enormous tumor expansion (Fig. 4, *B* and *C*,) without detectable change in Ki67 index (Fig. 4*D*). There could be several explanations for this phenotype. The GPCR domain of CD97 in H436A mutant cannot be activated by its built-in agonist and so the receptor may not internalize and would likely remain at the cell surface. The persistent basal activity of such a receptor may provide a low signaling output that could be sufficient to drive long-term tumor expansion. Moreover, the increased amount of these mutant receptors on the cell surface would also increase abundance of its covalently attached NTF, promoting cell survival through interactions with integrins (53). Furthermore, studies have shown that in prostate cancer, CD97 forms dimers with the lysophosphatidic acid receptors, which are also coupled to Gα_12/13_ and enhance its downstream signaling (17). Such interactions may contribute to the increased growth of the xenograft tumors in our experiments.

Finally, the dissociated NTF of CD97, often called a soluble CD97, itself may act as a ligand for the receptors on the surface of other cells (24, 52). In H436A tumors, this ligand could potentially bind and attract the host cells, such as tumor-associated macrophages or other stromal cells, also contributing to tumor volume increase.

We conclude that overall, CD97 has limited effect on proliferation but rather, may affect viability of tumor cells. The extracellular NTF domain of CD97 plays an important role in GBM tumor expansion, possibly through several mechanisms.

### CD97 in tumor invasiveness

The uniquely high invasiveness of GBM is a major obstacle for its successful treatment. Our *in vitro* experiments confirmed that the changes in CD97 expression levels affect the invasive behavior of primary pdGSCs. In addition, we demonstrated that manipulation of CD97 in these cells caused differential secretion of soluble factors that modify the integrity of AJs between endothelial cells (Fig. 3). Interestingly, the secretion of these factors occurred when GBM cells had reduced levels of CD97 (the knockdown) or the activity of the receptor was inhibited by the H436A mutation. These data suggest that the presence of active CD97 attenuates secretion of such factors. Yet, we observed an increased invasion of the GBM cells with CD97 knockdown *in vitro* and *in vivo* (Figs. 1*D* and Figure 5, Figure 6, Figure 7). It is plausible to speculate that the observed increase in factor secretion is an adaptive response of pdGSCs to the reduction in CD97 levels and/or its activity.

Plasticity of cancer stem cells and significant inter- and intra-heterogeneity of GBM stem cells are well documented (1, 4, 56). Genetic profiling revealed that GBM tumor from the same patient can contain neoplastic cells with different unique genetic signatures that with time may morph to a different stem cell subpopulation (1). Interestingly, other studies have revealed that unlike highly diverse GSCs from tumor core, infiltrating GBM cells are more homogeneous and share common genetic characteristics (57). These infiltrating cells express, among others, genes involved in apoptosis resistance, regulation of cell-cell adhesion, and CNS development. It has been proposed that these invading cells exploit CNS developmental processes, allowing them to migrate over long distances. This migration is facilitated by epithelial-to-mesenchymal transition and is defined as single cell invasion (32). Notably, this invasion pattern differs from collective invasion, where tumor mass extends protrusions composed of tumor cells connected by cell-to-cell adhesion (30).

We found that CD97 knockdown and expression of the H436A mutant produced distinct invasion patterns of pdGSCs xenografts. Based on our results with CD97 knockdown tumors, we propose that the majority of pdGSCs in these tumors do not survive, but the surviving cells exhibit a robust single cell invasive phenotype. This hypothesis is supported by our experiments with noninvasive U87 cells (Fig. 5*E*). Indeed, after CD97 knockdown, a small population of these cells acquired a single cell invasive phenotype possibly through the change of their genetic profile. We detected much smaller number of infiltrating cells in U87 knockdown tumors likely because this cell line is more genetically uniform than the primary cells. In contrast, expression of the noncleavable H436A CD97 mutant reduced the number of individually infiltrating cells and facilitated a collective pattern of invasion with massive cell-packed protrusions. These results indicate that inability of NTF to dissociate causes strong cell-to-cell adhesion promoting this type of tumor invasion.

The H436A mutant is unable to stimulate Gα_12/13_ signaling (22). However, it is likely that other proinvasion pathways can still be activated upon overexpression of this mutant, for example, through the autocrine function of NTF on other cell surface receptors (17). Reciprocally, the deletion of NTF (ΔNTF) constitutively activates the Gα_12/13_ signaling that is known to promote cell motility *via* Rho pathway (27, 54). Accordingly, single cells from the ΔNTF-expressing tumors retain the ability to migrate along CC and to infiltrate the brain tissue (Figs. 6 and 7). Furthermore, we found that the ΔNTF tumors had much looser core structure and diffused edges indicative of cell infiltration. This finding is consistent with the idea that NTF interaction with integrins results in their upregulation and promotes cell adhesion (53). However, the irreversible NTF dissociation and activation of this GPCR can weaken this adhesion and promote tissue invasion by individual tumor cells.

### The dual role of CD97

**Figure 8:**
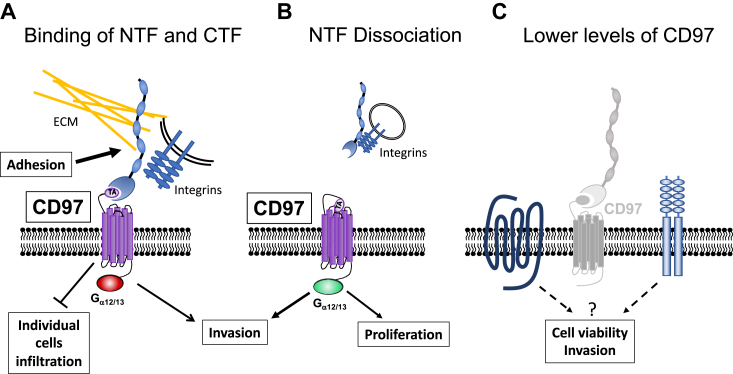
**The proposed model of the CD97 function in GBM tumor.***A*, the noncovalent binding of two CD97 functional domains inhibits receptor activation of its downstream signaling. In this state, CD97 promotes cell-to-cell or cell-to-ECM adhesion *via* NTF interaction with other receptors and the components of ECM. These interactions enhance collective tumor invasion but attenuate infiltration of individual tumor cells into the brain tissue. *B*, complete dissociation of the two functional CD97 fragments is irreversible and activates downstream signaling pathways mediated by Gα_12/13_, such as the Rho pathway, promoting proliferation and cell motility. The dissociated NTF, known as soluble CD97, acts as a ligand for receptors on the cell surfaces of other cells. *C*, GSCs with significantly decreased CD97 expression could be susceptible to cell death in the tumor environment. The high plasticity of GSCs enables survival of some cells with reduced levels of CD97 and triggers their transformation to a highly invasive phenotype mediated by the activity of other receptor(s). ECM, extracellular matrix; GBM, glioblastoma; NTF, N-terminal fragment.

In summary, we can draw several conclusions regarding the role of CD97 in GBM and propose the following model (Fig. 8). The linkage of NTF with the GPCR moiety of CD97 enhances cell-to-cell adhesion and facilitates collective invasion of the GBM tumors while inhibiting individual cell motility and infiltration. The NTF detachment leads to activation of Rho-mediated growth-promoting pathways that may increase tumor cell proliferation and tumor growth. Lower levels of CD97 can reduce cell viability but may also cause a transformation of surviving cells toward a highly invasive phenotype. These insights into the role of CD97 domains will be important for evaluating the potential of this aGPCR as a drug target in the treatment of GBM.

## Experimental procedures

### Materials and reagents

The antibodies used include anti-human GAPDH (Sigma-Aldrich, Cat# HPA061280, RRID:AB_2684463), VE-Cadherin (Sigma, Cat# MABT134), CD97 (Abcam, Cat# ab108368), mouse FLAG (Cell Signaling Technology, Cat# 8146; RRID:AB_10950495), rabbit FLAG (Cell Signaling Technology, Cat# 14793), GAPDH (Thermo Fisher Scientific, Cat# AM4300, RRID:AB_2536381), rabbit anti-human GAPDH (Sigma-Aldrich, Cat#ZRB374), Ki67 (Thermo Fisher Scientific, Cat# RM-9106, RRID:AB_2341197). All secondary antibodies were conjugated to Alexa Fluor dyes purchased from Jackson Immuno Research. Growth factor–reduced Matrigel matrix, (Corning, Cat# 354230), chondroitin sulfate (#230699), and hyaluronic acid (H7630) were used for invasion assay at specified concentrations. To culture primary GBM cells, vessels were coated with 0.1 mg/ml poly D-lysine hydrobromide (Sigma, Cat# P7886) and 0.01 mg/ml laminin (Corning, Cat# 354232). HUVEC cells (Lonza, Cat# C2519A) were cultured in flasks coated with 0.1% gelatin (Sigma, Cat# G9391). HUVEC authentication was done by the supplier with double immunostaining for CD31/CD105 markers and was more than 90% positive. Cells were lifted with either cell stripper (Corning, Cat# 23-25-056-CI), accutase (Sigma, Cat# A6964), or TrypLE (Gibco, Cat# 1349171).

### Surgical specimens and primary GSC culture

Primary GSC lines were established from patient specimens as previously described (35), and tissue was collected according to IRB #20190521. The dissociated cells were plated on Poly-D-Lysine (PDL) (0.1 mg/ml)/laminin (10 μg/ml) coated flasks and cultured at 37 °C 5% CO_2_ in GBM media with the following composition: DMEM/F12 + GlutaMAX (Gibco, Cat #, 10565-018) supplemented with B27 (Life Technologies, Cat# 17504-044), N2 (Thermo Fisher Scientific, Cat# 17502001), 1% penicillin/streptomycin (Life Technologies #15140-122), 1 mM Na pyruvate (Life Technologies, Cat# 11360-070), 20 ng/ml epidermal growth factor (Stem Cell Technologies, Cat# 78006), 20 ng/ml basic fibroblast growth factor (Stem Cell Technologies, Cat # 78003), and 1 IU/ml heparin (Stem Cell Technologies, Cat# 07980). The tissue specimens from GBM 1, 12, and 22 patients were all confirmed to be GBM by a neuropathologist. The genetic analysis identified their mutation status and confirmed that all tumors were IDHwt and MGMT nonmethylated. This data was reported in the previous publication (35) that also characterized the pdGSCs by the expression of GBM subtype markers designating GBM1 and 22 to mesenchymal and GBM12 to proneural subtypes.

### Plasmid constructs and lentivirus production

shRNAs for CD97 (target sequence 5′ACAAGAAGGTTCGGGAAGAAT) and control (5′-CGTACGCGGAATACTTCGA) were cloned into the pLKO-Puro-TRC plasmid (Addgene, Cat# 10878; RRID: Addgene_10878), following the Addgene protocol for pLKO plasmid. To trace cells expressing shRNAs, GFP was introduced by PCR amplification of GFP-IRES fragment from the HIV-GFP-Luc plasmid (Addgene, Cat# 21375; RRID: Addgene_21375) and cloned into the BamHI site of pLKO-Puro-TRC. Flag-tagged CD97 cDNA was PCR-amplified from CD97-Tango (Addgene, Cat# 66247; RRID: Addgene_66247) and cloned into pLenti-CMV-Puro (Addgene, Cat# 17448) to replace the GFP cDNA. To create the noncleavable H436A mutant, “CA” nucleotides were changed to “GC” to replace His with Ala amino acid in the “HLS” sequence at CD97 GPS. The ΔNTF truncated mutant was constructed by amplifying the CTF of CD97, starting from “SSF…” amino acid sequence and introducing methionine at the start. This fragment was cloned into the SalI site of the pCMV-GFP-Puro plasmid (Addgene, Cat# 17448; RRID:Addgene_17448) in-frame with GFP. The resulting plasmid produced the CD97-CTF-GFP fusion protein and includes the TA. In subsequent experiments, this CD97 mutant named as ΔNTF.

A second generation lentivirus was produced according to the Addgene-suggested protocol using psPAX2 (Addgene, #12260) and pMD2.G (Addgene, #12259) as helper plasmids. Briefly, the transfer and two helper plasmids were transfected into HEK293T cells (ATCC, Cat#CRL-3216, RRID: CVCL_0063) using PEI at 1 mg/ml (Sigma, Cat# 764604) as a transfection agent. The released virus was harvested twice and filtered through 0.45 μm filter. The virus was concentrated using a Viro-Peg Concentrator (Thomas Scientific, Cat# CHM00B952), and aliquots were stored at −80 °C. The viral titer was tested using serial dilutions of reporter-positive constructs. Puromycin (1 μg/ml) was used for at least 5 days to select transduced GBM cells.

### Testing Rho pathway activation in HEK239 cells using luciferase reporter plasmid

pGL4.34 vector (Promega, [luc2P/SRF-RE/Hygro], #E1359) encoding the luciferase driven by Serum Response Factor–Response Element promoter was used as a reporter to detect the activation of the Rho pathway. HEK293 cells were plated in the black opaque 96-well plate (Corning, #3916) at 10,000 cells/well and let to adhere for at least 5 h. For background control, one raw of wells contained only media. pGL4.34 was cotransfected with the plasmids encoding GFP (also used as an “empty” plasmid), flag-tagged CD97, or ΔNTF mutant that has GFP at its C terminus cloned in-frame. Plasmid expressing constitutively active RhoA (Addgene, # 61632) was used as a positive control, and the plasmid encoding RGS domain of p115-Rho-GEF (Addgene, # 112935) was used to confirm the specificity of Rho pathway activation. FuGENE transfection reagent (Promega, #E5911) was used to deliver the plasmid DNA into HEK293 cells. For transfection, the 1:3 DNA to FuGENE ratio was used, with 1 μg of total DNA to 3 μl of FuGENE in 100 μl of OptiMEM serum-reduced media (Gibco, #31985070). The following amounts of plasmids were used: pGL4.34 to 400 ng, tested CD97 constructs – 300 ng, and controls – 300 ng. The DNA:FuGENE:OptiMEM mix was incubated 15 min at room temperature and then 20 μl per well added to the plated HEK293 cells. Cells were left in the CO_2_ incubator for about 16 h followed by media replacement with 80 μl of the fresh DMEM/10% FBS. To determine the numbers of viable cells to each well, 20 μl of 5× CellTiter-Fluor solution (Promega, #G6080) was added. The plate was briefly mixed and incubated for another 30 min at 37 °C in CO_2_ incubator followed by the measurement of fluorescence at 380-400nm_Ex_/505nm_Em_. For each value of the measured fluorescence, we subtracted the average value of the recorded background wells. Hundred microliters of One-Glo Luciferase (Promega, #E6120) assay solution per well was added following the fluorescence measurement. The plate was placed on a rotating platform to mix for 3 min at room temperature following the measurement of luminescence at 0.5 s/well. The recorded value of the luminescence for each well was divided to the obtained corresponding fluorescence value to normalize for cell viability. The results were plotted on the graphs as a mean ± SD

### Western blot

Cells were lifted and resuspended in RIPA buffer (50 mM Tris–HCL, 150 mM NaCl, 1.0%(v/v) NP-40, 1.0%(w/v) Na deoxycholate, 1.0 mM EDTA, 0.1% SDS, 0.01% Na Azide, pH 7.4) supplemented with protease inhibitor cocktail (04693124001, Roche). After incubation for 15 min on ice, lysates were centrifuged for 15 min at 4 °C and the supernatant saved. Protein concentration was measured using bicinchoninic acid assay (Thermo Fisher Scientific, Cat# 23227) according to the manufacturer’s instructions. Supernatant was mixed with 4× SDS loading buffer (200 mM Tris–HCL pH 6.8, 4% SDS, 40% glycerol, bromophenol blue, 10% β-mercaptoethanol) and boiled for 5 min. Lysate boiling was omitted for detection of CD97 receptor by Western blot. Lysates were aliquoted and stored at −80 °C. Imaging acquisition was done on Azure Biosystem C600 using fluorescently labeled secondary antimouse antibodies conjugated with IRDye680RD (LiCor, Cat# 926-68070) and anti-rabbit SW800 (Invitrogen, Cat# SA5-10036).

### Invasion assays

For each invasion assay, transduced cultured pdGSCs were selected with puromycin (1 μg/ml) for at least 5 days before starting the experiment. RTCA invasion assay in two chamber CIM plates (Agilent, cat# 5665817001) was done using xCelligence instrument (Agilent, RTCA-DP) as previously described (35) with slight modifications. Briefly, ice-cold 50 μl of Matrigel supplemented with chondroitin sulfate (20 mg/ml) and hyaluronic acid (10 μg/ml) was layered to the bottom of the cold CIM plate upper chamber. Twenty microliters of the Matrigel were then taken out from each well and discarded. The chamber was incubated at 37 °C CO_2_ incubator for at least 4 h or overnight. The wells of the lower chamber were filled with 160 μl of GBM growth media supplemented with 10% FBS. Once the Matrigel was set, the upper and the lower chambers were assembled by snapping them together and 50 μl of GBM growth media (without FBS) was added to each well of the upper chamber. The assembled plate was placed onto the xCelligence instrument inside CO_2_ incubator and equilibrated for 1 hour. The background measurement of the equilibrated plate was taken before adding the cells. The modified pdGSCs were lifted, counted, and resuspended in GBM media at 3 x 10^4^-5x10^4^ cells/100 μl. Hundred microliters of each cell suspension was plated into corresponding well of the assembled CIM plate and placed back in the incubator into the cradle of the instrument. For each condition, 4 to 6 technical replicas were plated.

The real-time data obtained by the instrument was plotted on the graph in arbitrary units expressed as the “cell index” measuring changes in impedance every 15 min. The cell index in this invasion assay is proportional to the number of cells crossing the Matrigel barrier from upper to the lower chamber. The traces of the cell index taken from the live cells over time reflect the ability of the cells to invade the matrix toward the attractant (FBS) showing higher numbers for more invasive cells.

For real-time invasion assay using HUVECs monolayer, we adapted the previously published protocol (37). In short, the HUVECs were plated at 2.5 × 10^4^ cells/well on E-Plate (Agilent, cat# 300600910) coated with 0.1% gelatin. The cells were cultured in HUVECs media (Lonza, Cat# CC-5035) in CO_2_ incubator at 37 °C for at least 16 h. The cell index would show the growing curve indicating the surface area occupied by the increasing number of adhering cells until the cell monolayer was formed. At this point, the cell index would not be changed showing the flat line. The modified pdGSCs were lifted and resuspended in GBM media at 1 x 10^4^ cell/100 μl. The HUVEC media was then replaced by 100 μl of pdGSCs suspensions in the corresponding wells of the E-Plate. Four to six technical replicas were done for each condition. To control for the media change effect, one set of replica wells with HUVECs was replaced with the HUVECs media and another set with GBM media without cells. The E-plate was then returned to CO_2_ incubator to monitor changing cell index for 24 to 48 h taking the measurements every 15 min. For data analysis, the cell index was normalized to the time of pdGSCs addition to HUVECs. The invading pdGSCs disrupt the dense monolayer causing HUVECs to round up reducing the occupied plate surface. Thus, the cell index in this assay will be reducing for more invasive GBM cells.

### Tight junction integrity and stability test

The pdGSCs stably transduced with lentiviruses carrying CD97 constructs were cultured in GBM growth media for 2 days. The media then was collected and centrifuged to remove any cell debris. GBM conditioned media was then mixed 1:1 with HUVECs media and added to a monolayer of HUVECs that were plated on round 18 mm glass cover slips coated with 0.1% gelatin in 12-well plate. The HUVECs were incubated with the conditioned media for 1 hour in CO_2_ incubator at 37 °C. The cells then were fixed and stained with VE-cadherin antibody and Phalloidin to identify endothelial junctions and filamentous actin, respectively. Andor Dragonfly 202 Confocal microscope (Andor Technology Ltd) was used to acquire the images at 40× magnification. Images were processed using ImageJ (ImageJ, RRID:SCR_003070). The experiment was repeated three times to produce three biological replicas. For cell imaging analysis, 3 to 4 visual fields were randomly taken per condition with 30 to 60 cells per each field. VE-Cadherin and phalloidin staining were used to identify the morphology of AJs between HUVECs. The images from all three experiments were coded and guided by the defined morphological characteristics, analyzed blindly to count each AJ type.

### Cells immunostaining

For confocal microscopy, primary GBM cells were plated on glass cover slips coated with PDL(100 μg/ml)/laminin(10 μg/ml). Cells were fixed for 25 min with 4% paraformaldehyde (PFA) at room temperature, washed with PBS, permeabilized with 0.5% Triton X-100 for 5 min, and washed again. Cells then were blocked in blocking buffer (5% normal goat serum, 1% bovine serum albumin, 0.03% Triton X-100 in PBS) for 1 h at room temperature. The primary antibodies were diluted in blocking buffer at required concentration, and cells were incubated overnight at 4 °C. Cells were washed with PBS and incubated with the secondary antibodies in the blocking buffer for 2 h at room temperature and washed again before mounting on the slide using Prolong Glass (LifeScience, Cat# P36982). Andor Dragonfly 202 Confocal microscope (Andor Technology Ltd) was used to acquire the images. Image analysis was done using ImageJ software (https://imagej.nih.gov/ij/index.html) (ImageJ, RRID:SCR_003070).

### Stereotactic injection of pdGSCs into mouse brain

Female athymic mice (Foxn1^nu^/Foxn1^nu^) (RRID:IMSR_JAX:007850) were housed under pathogen-free conditions and received food and water *ad libitum*. The mice were 6-7 weeks old at the time of surgery. All mouse surgeries were done according to IACUC guidelines and approved by the University of Miami IACUC commission. Before the surgery, the mice were anesthetized with ketamine(10 mg/ml)/xylazine(1 mg/ml) solution at ketamine dose of 100 mg/kg.

Primary pdGSCs were undergoing the following preparation prior to brain implantation. Cells were plated at 70% confluency in 6-well plates coated with PDL(0.1 mg/ml) and laminin(10 μg/ml). After cell attachment, 40 μl of each concentrated lentiviral stock was added to corresponding well and left overnight in CO_2_ incubator at 37 °C. Next day, the media was changed, and cells were cultured for two more days. Three days post infection, the transduced cells were put in selection media containing 1 μg/ml of puromycin and cultured in this media for 1 week with two media changes to remove dead cells. At the day of the surgery, puromycin-selected cells were harvested with cell stripper or accutase, and 1 × 10^5^ cells resuspended in 2 μl of PBS were injected using a stereotactic frame into prefrontal lobe at the following coordinates—1 mm anterior, 1.5 mm to the right of Bregma, at 3 mm depth using a Hamilton syringe (Hamilton 80366) into five mice per condition. These coordinates assured the tumor formation at a specific position that avoids ventricles and also crosses CC. The mice were injected with 30 μl of buprenorphine SR (1 mg/ml) for analgesia and monitored for postsurgery recovery for at least 3 days. The tumor growth was monitored using IVIS noninvasive imaging system at University of Miami Cancer Center animal facility.

### Mouse brain harvesting and embedding

The previously established average survival time for mice transplanted with GBM1 or GBM22 cells was to be approximately 8 weeks. Therefore, to ensure consistency in comparison of the xenografts formed by modified pdGSCs, the brains were harvested after 6 weeks after implantation procedure. Mice were euthanized with an overdose of ketamine(50 mg/ml)/xylazine(20 mg/ml) 1:1 mixture and perfused with PBS following 4% PFA. Extracted mice brains were further fixed in 4% PFA overnight followed by extensive PBS washing to remove the fixative. Brains were then equilibrated in 30% sucrose before embedding in the freezing matrix (Thermo Fisher Scientific, M-1 Embedding Matrix- REF 1310). Brains were sliced coronally using a cryostat (Leica CM1900) set to 20-micron thickness and placed on positively charged microscope slide (Surgipath X-tra, Leica #38800200). Five coronal brain sections were placed on each slide in such an order that neighboring sections will be in each horizontal row among five consecutive slides. Therefore, each slide had the brain slices spaced 100 microns apart and together cover the 500 microns tissue thickness. To ensure the inclusion of the whole tumor spread, for each mouse brain, 50 slides were collected containing 250 tissue sections covering almost the entire brain.

### Immunostaining of brain tissue sections

Frozen brains sections were defrosted and washed two times with PBS for 10 min to remove excess freezing media. Slides were then baked at 60 °C for 30 min followed by antigen retrieval in sodium citrate buffer (10 mM sodium citrate, 0.05% Tween 20, pH 6.0) at 90 °C for 5 min. Slides were then placed into cassettes (Coverplate, Thermo Fisher Scientific, #72110017), washed with PBS, permeabilized with 1% Triton X-100 in PBS for 15 min, and washed again. The tissues were incubated in the blocking buffer (BB) (5% normal goat serum, 2% bovine serum albumin, 0.3% Triton X-100 in PBS) for 4 h at room temperature. The primary antibodies were diluted in BB at required concentration, and slides were incubated overnight at 4 °C. Brain sections were washed with 0.05% Tween 20 in PBS and incubated with the secondary antibodies in the BB for 2 h at room temperature. Slides were washed again in 0.05% Tween 20 in PBS, removed from cassettes, coated with mounting media, and sealed.

### Imaging and analysis of brain sections

VS120 Slide Scanner microscope (Olympus LS) was used to acquire images that were processed using OlyVIA software, version 2.9 (Olympus LS, Japan) (https://www.olympus-lifescience.com/en/discovery/image-sharing-made-easy-meet-olyvia/). ImageJ software (ImageJ, RRID:SCR_003070) was used for image analysis to calculate tumor cells invasion and proliferation. Three mice were randomly selected for each experimental condition. For each mouse, 5 to 6 slides around injection site were selected and scanned on a slide scanner. Each slide produced five images of the entire brain sections at 10× magnification. Аn estimate of up to ninety images were analyzed per condition, reflecting the analysis of 25 to 30 images for each of the three mice in each condition. Quantitative analyses of tumor cells migrating along CC and proliferating cells in tumor bulk were done using macros written in ImageJ.

To analyze tumor proliferation, brain sections were stained with Ki67 antibodies to identify proliferating nuclei and with DAPI to label all nuclei. FLAG staining and GFP fluorescence were used to mark cells expressing the construct. One slide from each three randomly selected mice was chosen, reflecting the analysis of 15 images for each experimental condition. Each slide contained five brain sections located close to the injection site to ensure the analysis of cell proliferation within the tumor core. The ROI was outlined close to the tumor center while avoiding the necrotic tissue, which was noticeable by the absence of fluorescence.

An ImageJ macro was created to identify and calculate several parameters: a) the total number of nuclei; b) the number of Ki67-positive nuclei; c) the number of Ki67-positive and total nuclei only in the GFP- or FLAG-positive cells. To quantify nuclei, the threshold was established for the DAPI and Ki67 channels and the particle size was set to above 10 μm to exclude nonspecific noise. To calculate the number of proliferating cells only in the GFP- and Flag-positive tumor areas, the masks were created using the threshold for green channel. The masks were then applied to Ki67 and DAPI channels producing the nuclei counts of only construct-positive cells. Using the ImageJ macro, all chosen images were processed automatically producing four numbers for each ROI: 1) the number of Ki67- and the number of DAPI-positive nuclei without applying the mask and 2) the Ki67- and DAPI-positive nuclei numbers only in the areas covered by the green channel mask. The ratios of Ki67-positive to total DAPI-positive nuclei expressed as a percentage were plotted to the graph, where each dot represented a calculated value in each ROI.

To quantify pdGSCs single cell invasion along CC, brain sections were stained with hGAPDH antibodies to differentiate human from mouse cells, FLAG antibodies to identify N terminus of CD97 in CD97-OE– or H436A-transduced cells (see Fig. 2), and GFP fluorescence to detect cells expressing WT, shCD97, and ΔNTF constructs. Two ImageJ macros were developed. One macro was designed to identify the area occupied by the tumor bulk and establish its borders. The other macro quantified the number of pdGSC migrating along CC.

The area occupied by the tumor mass was calculated as a percentage relative to the entire area of the brain section (Fig. 4*C*). For these calculations, we used 10 to 12 brain sections around the injection site covering about 0.5 mm of the brain thickness. To outline the tumor bulk using ImageJ, the measurement parameters were setup to identify the densest growth of the tumor cells excluding the very small cell clusters by size. The tumor bulk area was identified by setting up the threshold for hGAPDH fluorescence and limiting the size of particles to no less than 1000 μm in diameter, the size of a small cell cluster. The use of this macro helped to identify the tumor edge and to calculate the area occupied by the tumor bulk. To quantify migrating single GBM cells along CC, in ImageJ, seven equal size ROIs were drawn along CC. The first ROI was placed at the distance equal to two ROI’s lengths starting at defined tumor edge. The analyzed brain sections were chosen to be near the injection site. Using the second macro, the threshold was setup in each ROI for hGAPDH and separately, for GFP or FLAG fluorescence. In this macro, the particle size was limited to 10 to 1000 μm to identify individual cells and small cell clusters. Of note, the fluorescence intensity of GFP or FLAG staining varied depending on the expressed construct type (e.g., free soluble GFP in WT and shCD97 vs GFP in ΔNTF fusion protein). Therefore, we adjusted the threshold accordingly. All images were then automatically processed by the two macro programs, and the cell count from each ROI was used to generate graphs showing the density of GFP- or FLAG-positive cells at various distances from the tumor border. To better visualize the migration of modified pdGSCs in the thickness of CC, the average number of cells positive for GFP or FLAG fluorescence was calculated for each ROI located at a particular distance from tumor border. The mean ± SEM were plotted in the bar graphs with the distance in μm plotted on X axes and the average ROIs cell number on Y axes.

### Statistical analysis

Statistical analysis was performed using the PRISM software (version 9) for Mac OS X or JMP Pro 16.0.0 (Statistical Analysis System, RRID:SCR_008567) with a significance of *p* < 0.05. To determine statistical difference of measured proportions of AJs of HUVECs, we used two-way ANOVA Tukey’s multiple comparison test. To compare the calculations of tumor sizes and Ki67 proliferation index, Brown-Forsythe and Welch ANOVA with Dunnet’s multiple comparisons tests were performed. Nonsignificant (ns), *p* > 0.05; ∗ *p* < 0.05; ∗∗ *p*< 0.01; ∗∗∗ *p* < 0.001; ∗∗∗∗ *p* <0.0001.

## Data availability

The data generated and analyzed in this study are provided in the text, figures, tables, and supplementary tables. Additional data required can be requested from the corresponding author.

## Consent for publication

No individual patient data is reported in this study. Ethical approval was obtained from the University of Miami Institutional Review Board for Human Research (IRB #20190521). Written and verbally informed preoperative consent was obtained from all patients.

## Supporting information

This article contains supporting information.

## Conflict of interest

Dr Michael Ivan reports being a consultant and receiving research funding from Medtronic and AstraZeneca. The other authors declare that they have no conflict of interest with the contents of this article.

## References

[1] Neftel C., Laffy J., Filbin M.G., Hara T., Shore M.E., Rahme G.J. (2019). An integrative model of cellular states, plasticity, and genetics for glioblastoma. Cell.

[2] Molina J.R., Hayashi Y., Stephens C., Georgescu M.M. (2010). Invasive glioblastoma cells acquire stemness and increased Akt activation. Neoplasia.

[3] Hatoum A., Mohammed R., Zakieh O. (2019). The unique invasiveness of glioblastoma and possible drug targets on extracellular matrix. Cancer Manag. Res..

[4] Dirkse A., Golebiewska A., Buder T., Nazarov P.V., Muller A., Poovathingal S. (2019). Stem cell-associated heterogeneity in Glioblastoma results from intrinsic tumor plasticity shaped by the microenvironment. Nat. Commun..

[5] Chakravarty D., Pedraza A.M., Cotari J., Liu A.H., Punko D., Kokroo A. (2017). EGFR and PDGFRA co-expression and heterodimerization in glioblastoma tumor sphere lines. Sci. Rep..

[6] Lee J.C., Vivanco I., Beroukhim R., Huang J.H., Feng W.L., DeBiasi R.M. (2006). Epidermal growth factor receptor activation in glioblastoma through novel missense mutations in the extracellular domain. PLoS Med..

[7] Mooney K.L., Choy W., Sidhu S., Pelargos P., Bui T.T., Voth B. (2016). The role of CD44 in glioblastoma multiforme. J. Clin. Neurosci..

[8] Fields G.B. (2019). Mechanisms of action of novel drugs targeting angiogenesis-promoting matrix metalloproteinases. Front. Immunol..

[9] Meldolesi J. (2016). Pharmacology of the cell/matrix form of adhesion. Pharmacol. Res..

[10] Schiffer D., Mellai M., Boldorini R., Bisogno I., Grifoni S., Corona C. (2018). The significance of chondroitin sulfate proteoglycan 4 (CSPG4) in human gliomas. Int. J. Mol. Sci..

[11] Silver D.J., Siebzehnrubl F.A., Schildts M.J., Yachnis A.T., Smith G.M., Smith A.A. (2013). Chondroitin sulfate proteoglycans potently inhibit invasion and serve as a central organizer of the brain tumor microenvironment. J. Neurosci..

[12] Teixeira A.F., Ten Dijke P., Zhu H.J. (2020). On-target anti-TGF-beta therapies are not succeeding in clinical cancer treatments: what are remaining challenges?. Front. Cell Dev. Biol..

[13] Chaudhary R., Morris R.J., Steinson E. (2021). The multifactorial roles of microglia and macrophages in the maintenance and progression of glioblastoma. J. Neuroimmunol.

[14] Gray J.X., Haino M., Roth M.J., Maguire J.E., Jensen P.N., Yarme A. (1996). CD97 is a processed, seven-transmembrane, heterodimeric receptor associated with inflammation. J. Immunol..

[15] Li C., Liu D.R., Li G.G., Wang H.H., Li X.W., Zhang W. (2015). CD97 promotes gastric cancer cell proliferation and invasion through exosome-mediated MAPK signaling pathway. World J. Gastroenterol..

[16] Liu D., Trojanowicz B., Ye L., Li C., Zhang L., Li X. (2012). The invasion and metastasis promotion role of CD97 small isoform in gastric carcinoma. PLoS One.

[17] Ward Y., Lake R., Yin J.J., Heger C.D., Raffeld M., Goldsmith P.K. (2011). LPA receptor heterodimerizes with CD97 to amplify LPA-initiated RHO-dependent signaling and invasion in prostate cancer cells. Cancer Res..

[18] He Z., Wu H., Jiao Y., Zheng J. (2015). Expression and prognostic value of CD97 and its ligand CD55 in pancreatic cancer. Oncol. Lett..

[19] Safaee M., Clark A.J., Oh M.C., Ivan M.E., Bloch O., Kaur G. (2013). Overexpression of CD97 confers an invasive phenotype in glioblastoma cells and is associated with decreased survival of glioblastoma patients. PLoS One.

[20] Safaee M.M., Wang E.J., Jain S., Chen J.S., Gill S., Zheng A.C. (2022). CD97 is associated with mitogenic pathway activation, metabolic reprogramming, and immune microenvironment changes in glioblastoma. Sci. Rep..

[21] Hamann J., Aust G., Arac D., Engel F.B., Formstone C., Fredriksson R. (2015). International union of basic and clinical pharmacology. XCIV. Adhesion G protein-coupled receptors. Pharmacol. Rev..

[22] Vizurraga A., Adhikari R., Yeung J., Yu M., Tall G.G. (2020). Mechanisms of adhesion G protein-coupled receptor activation. J. Biol. Chem..

[23] Hamann J., Vogel B., van Schijndel G.M., van Lier R.A. (1996). The seven-span transmembrane receptor CD97 has a cellular ligand (CD55, DAF). J. Exp. Med..

[24] Wang T., Ward Y., Tian L., Lake R., Guedez L., Stetler-Stevenson W.G. (2005). CD97, an adhesion receptor on inflammatory cells, stimulates angiogenesis through binding integrin counterreceptors on endothelial cells. Blood.

[25] Wandel E., Saalbach A., Sittig D., Gebhardt C., Aust G. (2012). Thy-1 (CD90) is an interacting partner for CD97 on activated endothelial cells. J. Immunol..

[26] Kwakkenbos M.J., Pouwels W., Matmati M., Stacey M., Lin H.H., Gordon S. (2005). Expression of the largest CD97 and EMR2 isoforms on leukocytes facilitates a specific interaction with chondroitin sulfate on B cells. J. Leukoc. Biol..

[27] Juneja J., Casey P.J. (2009). Role of G12 proteins in oncogenesis and metastasis. Br. J. Pharmacol..

[28] Hsiao C.C., Wang W.C., Kuo W.L., Chen H.Y., Chen T.C., Hamann J. (2014). CD97 inhibits cell migration in human fibrosarcoma cells by modulating TIMP-2/MT1- MMP/MMP-2 activity--role of GPS autoproteolysis and functional cooperation between the N- and C-terminal fragments. FEBS J..

[29] Sharifi G., Pajavand A.M., Nateghinia S., Meybodi T.E., Hasooni H. (2019). Glioma migration through the corpus callosum and the brainstem detected by diffusion and magnetic resonance imaging: initial findings. Front. Hum. Neurosci..

[30] Friedl P., Locker J., Sahai E., Segall J.E. (2012). Classifying collective cancer cell invasion. Nat. Cell Biol..

[31] Friedl P., Wolf K. (2010). Plasticity of cell migration: a multiscale tuning model. J. Cell Biol..

[32] van Zijl F., Krupitza G., Mikulits W. (2011). Initial steps of metastasis: cell invasion and endothelial transmigration. Mutat. Res..

[33] Krakhmal N.V., Zavyalova M.V., Denisov E.V., Vtorushin S.V., Perelmuter V.M. (2015). Cancer invasion: patterns and mechanisms. Acta Naturae.

[34] Chien C.H., Hsueh W.T., Chuang J.Y., Chang K.Y. (2021). Dissecting the mechanism of temozolomide resistance and its association with the regulatory roles of intracellular reactive oxygen species in glioblastoma. J. Biomed. Sci..

[35] Eichberg D.G., Slepak T.I., Pascoini A.L., Komotar R.J., Ivan M.E. (2021). Genetic manipulation of adhesion GPCR CD97/ADGRE5 modulates invasion in patient-derived glioma stem cells. J. Neurooncol..

[36] Yosef G., Arkadash V., Papo N. (2018). Targeting the MMP-14/MMP-2/integrin alphavbeta3 axis with multispecific N-TIMP2-based antagonists for cancer therapy. J. Biol. Chem..

[37] Rahim S., Uren A. (2011). A real-time electrical impedance based technique to measure invasion of endothelial cell monolayer by cancer cells. J. Vis. Exp..

[38] Giannotta M., Trani M., Dejana E. (2013). VE-cadherin and endothelial adherens junctions: active guardians of vascular integrity. Dev. Cell.

[39] Ward Y., Lake R., Faraji F., Sperger J., Martin P., Gilliard C. (2018). Platelets promote metastasis *via* binding tumor CD97 leading to bidirectional signaling that coordinates transendothelial migration. Cell Rep..

[40] Hambardzumyan D., Gutmann D.H., Kettenmann H. (2016). The role of microglia and macrophages in glioma maintenance and progression. Nat. Neurosci..

[41] Cheng Z., Garvin D., Paguio A., Stecha P., Wood K., Fan F. (2010). Luciferase reporter assay system for deciphering GPCR pathways. Curr. Chem. Genomics.

[42] Huveneers S., Oldenburg J., Spanjaard E., van der Krogt G., Grigoriev I., Akhmanova A. (2012). Vinculin associates with endothelial VE-cadherin junctions to control force-dependent remodeling. J. Cell Biol..

[43] Fernandez-Martin L., Marcos-Ramiro B., Bigarella C.L., Graupera M., Cain R.J., Reglero-Real N. (2012). Crosstalk between reticular adherens junctions and platelet endothelial cell adhesion molecule-1 regulates endothelial barrier function. Arterioscler. Thromb. Vasc. Biol..

[44] Kroeze W.K., Sassano M.F., Huang X.P., Lansu K., McCorvy J.D., Giguere P.M. (2015). PRESTO-Tango as an open-source resource for interrogation of the druggable human GPCRome. Nat. Struct. Mol. Biol..

[45] Cribaro G.P., Saavedra-Lopez E., Romarate L., Mitxitorena I., Diaz L.R., Casanova P.V. (2021). Three-dimensional vascular microenvironment landscape in human glioblastoma. Acta Neuropathol. Commun..

[46] Chidambaram A., Fillmore H.L., Van Meter T.E., Dumur C.I., Broaddus W.C. (2012). Novel report of expression and function of CD97 in malignant gliomas: correlation with Wilms tumor 1 expression and glioma cell invasiveness. J. Neurosurg..

[47] Galle J., Sittig D., Hanisch I., Wobus M., Wandel E., Loeffler M. (2006). Individual cell-based models of tumor-environment interactions: multiple effects of CD97 on tumor invasion. Am. J. Pathol..

[48] Huang L.C., Hueng D.Y. (2014). CD97 and glioma invasion. J. Neurosurg..

[49] Yin Y., Xu X., Tang J., Zhang W., Zhangyuan G., Ji J. (2018). CD97 promotes tumor aggressiveness through the traditional G protein-coupled receptor-mediated signaling in hepatocellular carcinoma. Hepatology.

[50] Tjong W.Y., Lin H.H. (2019). The role of the RGD motif in CD97/ADGRE5-and EMR2/ADGRE2-modulated tumor angiogenesis. Biochem. Biophys. Res. Commun..

[51] Liu D., Li C., Trojanowicz B., Li X., Shi D., Zhan C. (2016). CD97 promotion of gastric carcinoma lymphatic metastasis is exosome dependent. Gastric Cancer.

[52] Aust G., Zheng L., Quaas M. (2022). To detach, migrate, adhere, and metastasize: CD97/ADGRE5 in cancer. Cells.

[53] Tjong W.Y., Lin H.H. (2019). The RGD motif is involved in CD97/ADGRE5-promoted cell adhesion and viability of HT1080 cells. Sci. Rep..

[54] Kelly P., Casey P.J., Meigs T.E. (2007). Biologic functions of the G12 subfamily of heterotrimeric g proteins: growth, migration, and metastasis. Biochemistry.

[55] Rajagopal S., Shenoy S.K. (2018). GPCR desensitization: acute and prolonged phases. Cell Signal..

[56] Verhaak R.G., Hoadley K.A., Purdom E., Wang V., Qi Y., Wilkerson M.D. (2010). Integrated genomic analysis identifies clinically relevant subtypes of glioblastoma characterized by abnormalities in PDGFRA, IDH1, EGFR, and NF1. Cancer Cell.

[57] Darmanis S., Sloan S.A., Croote D., Mignardi M., Chernikova S., Samghababi P. (2017). Single-cell RNA-seq analysis of infiltrating neoplastic cells at the migrating front of human glioblastoma. Cell Rep..

