# Supplementary material for: Dual role of the adhesion G-protein coupled receptor ADRGE5/CD97 in glioblastoma invasion and proliferation
Source: J Biol Chem. 2023 Jul 28;299(9):105105. doi: 10.1016/j.jbc.2023.105105 (PMC10481366; doi:10.1016/j.jbc.2023.105105)
Supplement: Supporting information [file mmc1.docx]

**Supporting Information**

**Dual Role of adhesion G-protein coupled receptor ADRGE5/CD97 in Glioblastoma Invasion and Proliferation**

Tatiana I. Slepak^1,2^, Manuela Guyot^1,2^, Winston Walters^1,2^, Daniel G. Eichberg^1^, Michael E. Ivan^1,2,3^

^1^ Department of Neurosurgery, University of Miami Hospital, University of Miami, Coral Gables, USA.

^2^ Sylvester Comprehensive Cancer Center, University of Miami, Coral Gables, USA.

^3^ Corresponding Author, [mivan@med.miami.edu](mailto:mivan@med.miami.edu),1321 N.W. 14th Street, West Building, Suite 306, Miami, FL, 33125, USA.

**Running Title:** Dual Role of CD97 in Glioblastoma

**Supporting Materials**

Includes supplementary figures and figure legends.

**Fig. S1**

**Fig.S1** **Verification of CD97 antibodies specificity in cells, and confirmation of CD97 knockdown in tissue**

**A.** Confocal images of HEK293, GBM22, and U87 cells were stained with anti-CD97 antibody (red), Wheat Germ Agglutinin (WGA) lectin to label cell membrane (green), and DAPI for nuclei labeling (blue). In primary GBM22 cells and U87 GBM cell line, strong puncta CD97 staining co-localized with WGA labeled cell membrane (arrows). Cytosolic CD97 immunostaining was also observed. HEK293 cells, serving as a negative control, revealed minimal non-specific background staining. Scale bar is 20μm

**B.** Confocal images of tumors formed by U87 cells that were transduced with control (U87-WT) and shCD97 (U87-shCD97) constructs, both expressing GFP (green). The brain sections containing tumors were stained with CD97 antibody (red) and the images were captured at the same exposure for comparison of CD97 fluorescence intensity. The images show a significant reduction of CD97 fluorescence in the tumors with CD97 knockdown. Scale bar is 20μm

**Fig. S2**

**Fig.S2 Testing the activity of CD97 constructs via Gα_12/13_-mediated induction of the Rho pathway.**

**A.** Serum Response Factor- Response Element (SRF-RE) promoter is activated by Gα_12/13_-mediated Rho pathway. HEK293 cells were co-transfected with SRF-RE-Luciferase reporter plasmid and plasmids expressing GFP (negative control), active RhoA (positive control), wild-type CD97 (CD97-OE), and ΔNTF, a constitutively active CD97 mutant. Expression of luciferase was measured in relative luminescence units (RLU) and the obtained values were normalized to the number of viable cells. Both tested CD97 constructs responded to Rho activation with constitutively active ΔNTF mutant producing stronger signal compared to non-modified CD97. N=8, Mean ± SD

**B.** To verify that Rho pathway activation is mediated through Gα_12/13_ when CD97 constructs are expressed, HEK293 cells were co-transfected as described in (A), with the addition of the plasmid encoding RGS-p115, a specific Gα_12/13_ inhibitor. The inclusion of RGS-p115 significantly reduced the luminescence signal generated by both CD97 constructs, indicating that these receptors specifically activate the Rho pathway via Gα_12/13_ signaling. N=8, Mean ± SD

**Fig. S3**

**Fig.S3 Tumor size and proliferation quantification of the GBM1 derived tumors modified by CD97 constructs.**

The tumors produced by GBM1 primary pdGSCs transduced with indicated CD97 constructs where assessed for their size **(A)** and proliferation **(B)** as described for GBM22 cells in Fig.5 and 6.

**A.** The GBM1 tumors formed by cells over-expressing CD97 or its mutants were significantly larger in size compared to WT control, while the knockdown tumors, as for GBM22, were significantly smaller. The H436A GBM1 tumors were significantly larger than tumors formed by ΔNTF-expressing cells but were comparable in size with CD97-OE derived tumors. One-way ANOVA with Dunnett’s T3 multiple comparison test, n.s = p>0.1, **p<0.01, ****p<0.0001; N=3 Mouse Brains (5 brain slices per condition for each mouse), N=12-15, Mean ± SD.

**B.** The calculations of the Ki67 proliferation index of GBM1 tumors revealed that only ΔNTF mutant caused significant increase in tumor cells proliferation, while expression of other constructs did not significantly affect tumors proliferation. N=15, Mean ± SD.

One-way ANOVA with Dunnett’s T3 multiple comparison test, n.s. p>0.1, *p<0.1, **p<0.01; N=15 (3 Mouse Brains x 5 brain slices per condition for each mouse), Mean ± SD

**Fig.S4**

**Fig.S4 Quantitation method of GBM cells migrating along Corpus Callosum.**

Five consecutives 20-micron brain sections adjacent to the injection site were used to calculate the migration of the GBM cells along corpus callosum. A representative mouse brain slice image stained with the hGAPDH antibody is shown. Seven same-sized square regions of interest (ROIs) were equally spaced along the corpus callosum covering the distance of about 5 mm. The first ROI was positioned close to the tumor edge (inset, black curved line) at approximately two ROIs lengths from the injection site (asterisk). Using ImageJ software, the threshold for each fluorescent channel was used to identify stained cells, and a particle size restriction (50-1000μm) was applied to count only individual cells or very small cell clusters (5-10 cells).

**Fig. S5**

**Fig.S5 Migration of pdGSCs along corpus callosum from tumors formed by modified GBM1 cells.**

The figure demonstrates the migration patterns of primary glioblastoma stem cells (pdGSCs) along the corpus callosum (CC) in tumors generated by modified GBM1 cells. The mouse brains transplanted with the cells were processed as described for Fig.4A and brain sections were analyzed for cell migration along CC as described in S.Fig.4, and Fig.5 and 6. hGAPDH antibody were used to identify all human cells (red); GFP fluorescence identifies WT, shCD97 and ΔNTF expressing pdGSCs, and FLAG antibody used to label NTF of CD97 receptor in CD97-OE and H436A expressing cells (green). Scale bar for low magnification images of brain slices is 2000μm; for enlarged boxed areas scale bar is 500μm.

The control GBM1 tumors (WT) and tumors formed by pdGSCs expressing the constitutively active CD97 mutant ΔNTF exhibited extensive cell migration along myelinated tracts of CC. The shCD97 knockdown tumors were very small but still generated pdGSCs capable of invading the CC, albeit in much lower numbers compared to the control tumors.

In GBM1 tumors formed by cells expressing CD97-OE or H436A, there was a limited presence of FLAG-positive pdGSCs migrating along the corpus callosum (CC). However, the number of hGAPDH-positive cells, particularly in CD97-OE cells, was significantly higher.

**Fig. S6**

**Fig.S6 Quantification of GBM1 cells migrating along CC**

The numbers of GBM1 pdGSCs modified by CD97 constructs were quantified as described for and Fig.5 and 6. The placements of regions of interest (ROIs) is illustrated in S.Fig.4

**A.** The graphs show the calculated raw numbers of migrating cells where each dot represents the cell number per ROI (N=75, Mean ± SD). The top graph (red) shows the numbers for hGAPDH-positive cells, the graph below shows the numbers of GFP- or FLAG-positive cells.

**B.** The graph illustrates the average number of GFP- or FLAG-positive cells per region of interest (ROI) along the corpus callosum (CC), representing the migration of cells expressing the respective constructs. N=75, Mean ± SEM. Only a few GBM1 cells expressing shCD97 were detected in the CC likely due to poor survival.

CD97-OE and H436A tumors exhibited a minimal presence of FLAG-positive cells, suggesting that the majority of cells migrating along CC do not possess the FLAG-tagged NTF.**Fig. S7**

**Fig.S7 CD97 knockdown cause U87 cells to invade deep into the brain tissue.**

**A.** Representative mouse brain slices with tumors formed by control U87 cells (U87-WT) expressing GFP showed that no GFP-positive invading U87 cells were detected outside the tumor.

**B.** In contrast to **(A),** mouse brain slice with tumor formed by GFP-positive U87 cells expressing CD97 shRNA (U87-shCD97) shows the presence of GFP-positive “streaks” (arrows) that are likely the U87 cells migrating away from the tumor bulk.

Scale Bar – 2000μm

Insets Scale Bar – 500μm

**Fig. S8**

**Fig.S8 Invasion of modified GBM1 tumors into brain parenchyma**

Representative brain sections with tumors formed around the injection site (white asterisks) are shown. Scale bar 2000μm. Boxes are drawn at the edge of the tumor bulk to demonstrate the invasion of tumor cells into parenchyma. The enlarged framed images under each brain section show the merge and single channel monochrome images of the boxed area. Scale bar 500 μm.

Harvested mouse brains with tumors produced by GBM1 cells transduced with GFP control (WT), shCD97 (shCD97), FLAG-tagged cDNA for wild-type CD97 (CD97-OE), NTF-truncated CD97 (△NTF), and FLAG-tagged non-cleavable CD97 (H436A) constructs. All slides were stained with anti-hGAPDH (red) to differentiate between human and mouse cells. Brain sections expressing CD97-OE and H436A were also stained with anti-Flag antibodies (green). WT, shCD97, and ΔNTF express GFP (green).

All the modified tumors exhibited invasiveness into the brain tissue, but there were distinct differences in their patterns of invasion. H436A tumors displayed finger-like projections (arrows) composed of dense grouped tumor cells, with few infiltrating individual FLAG-positive cells. Similar projections were observed in CD97-OE tumors. However, in contrast to H436A tumors, tumors formed by CD97-OE cells showed a gradient of green fluorescence around tumor border (arrowheads), likely originating from the dissociated soluble CD97, which is the FLAG-tagged NTF of CD97. This fluorescence was not detected in tumors expressing non-cleavable H436A mutant.
